# Construction of Histone–Protein Complex Structures by Peptide Growing

**DOI:** 10.3390/ijms241813831

**Published:** 2023-09-07

**Authors:** Balázs Zoltán Zsidó, Bayartsetseg Bayarsaikhan, Rita Börzsei, Csaba Hetényi

**Affiliations:** Pharmacoinformatics Unit, Department of Pharmacology and Pharmacotherapy, Medical School, University of Pécs, Szigeti Út 12, 7624 Pécs, Hungary; zsido.balazs@pte.hu (B.Z.Z.); bayartsetseg704@yahoo.com (B.B.); rita.borzsei@aok.pte.hu (R.B.)

**Keywords:** docking, histone, peptide, ligand, fragment, growing

## Abstract

The structures of histone complexes are master keys to epigenetics. Linear histone peptide tails often bind to shallow pockets of reader proteins via weak interactions, rendering their structure determination challenging. In the present study, a new protocol, PepGrow, is introduced. PepGrow uses docked histone fragments as seeds and grows the full peptide tails in the reader-binding pocket, producing atomic-resolution structures of histone–reader complexes. PepGrow is able to handle the flexibility of histone peptides, and it is demonstrated to be more efficient than linking pre-docked peptide fragments. The new protocol combines the advantages of popular program packages and allows fast generation of solution structures. AutoDock, a force-field-based program, is used to supply the docked peptide fragments used as structural seeds, and the building algorithm of Modeller is adopted and tested as a peptide growing engine. The performance of PepGrow is compared to ten other docking methods, and it is concluded that in situ growing of a ligand from a seed is a viable strategy for the production of complex structures of histone peptides at atomic resolution.

## 1. Introduction

Histones have a diverse interaction profile [1] and play a key role in epigenetic regulation via interactions with the DNA in the chromatin [2,3], as well as various protein partners [4,5]. Readers are important proteins that distinguish between the combinatorial numbers of post-translationally modified histone molecules commonly called as the “histone code” [6]. The atomic-resolution structures of histone–reader complexes are key to understanding the “histone code” and designing new drugs that affect epigenetic regulation [6,7,8]. The present study is focused on consisting of histone H3 peptides and their reader proteins, which play an important role in the pathophysiology of various autoimmune diseases, intellectual disabilities, cancer development, such as breast cancer, colorectal carcinoma and hematopoietic cancers, autoimmune polyendocrinopathy–candidiasis–ectodermal dystrophy, meiotic defects in spermatocytes, breast, prostate and colorectal cancers, and leukemia (Table S1 [9,10,11,12,13,14,15,16,17,18]). These pathophysiological involvements render histone reader proteins such as bromodomains [19] and the eleven–nineteen leukemia protein (ENL [20]) attractive targets for drug design purposes.

**Figure 1:**
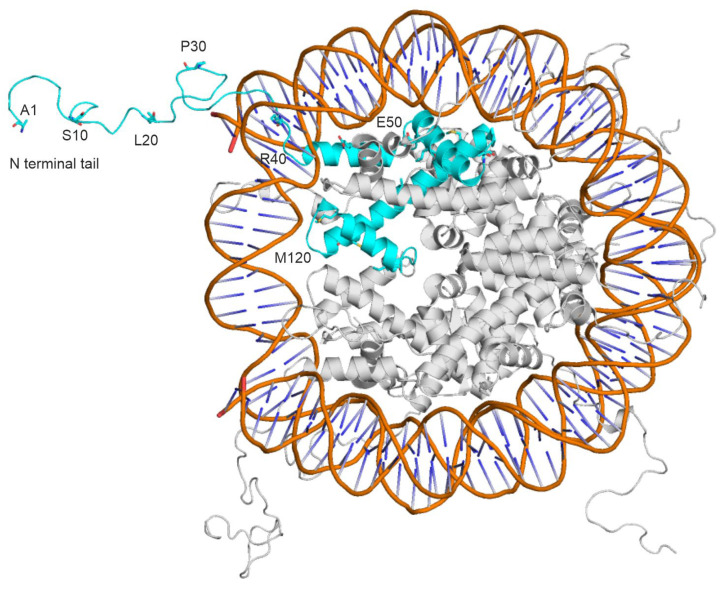
The terminal tails of histone proteins (teal and grey) stick out of the nucleosome core unit and have a flexible structure. The DNA backbone is colored in orange, base pairs are shown as dark blue sticks. A histone H3 protein is highlighted in teal. Every 10th amino acid of the histone H3 (teal) is marked. The figure was prepared from the PDB structure [39] 1kx5 using PyMol v2.0 [40].

While knowledge of the structures of histone H3–reader complexes is necessary for understanding the pathomechanism of epigenetic diseases and designing new drugs to act against them, the determination of their atomic-resolution structures can be rather challenging [21]. Experimental difficulties are presented by the creation of well-diffracting and stable crystals in X-ray crystallography [22], the computational processing of noisy images in cryo-electron microscopy [23], and the isotopic labeling of proteins in NMR [24]. Histones are particularly problematic ligands for structural determination, as they have a linear N-terminal tail with a high degree of conformational flexibility [25,26,27] that sticks out of the nucleosome structure (Figure 1). The protruding N-terminal tails of histones may interact with histone readers (like the proteins in Table S1) or with DNA [28]. Thus, the binding of the N-terminal tail of histone H3 with DNA may compete with the binding of histone N-terminal tails to histone reader proteins [29], which is further supported by the increased accessibility of histone H3 during nucleosome disassembly during transcription [30]. Like all peptides, histones are also extensively hydrated, which further complicates the determination of their interactions [5,31]. Moreover, there are shallow binding pockets on the reader side that result in the histone–reader complexes possessing moderate stability [32,33], with micromolar binding constants (see K_d_ values in Table S1 for examples). Long peptides such as histone tails are well-known problematic cases for fast computational docking [31,34], due to the inappropriateness of the scoring schemes [35,36,37] of their binding modes (position, orientation, and conformation) and the lack of explicit water models [38]. The complexes presented in Table S1 are good representatives for investigations of the above structural challenges.

The recognition of the above structural and methodological challenges accelerated the development of numerous fast docking methods for peptide ligands. At least three branches can be distinguished among the different methods: physico-chemical approaches, knowledge-based approaches, and their hybrid [41]. Physico-chemical approaches [42,43,44] calculate energy (scoring) values directly from the atomic positions of the molecules, without conducting further training or experiments. Knowledge-based methods [34,41] are relatively fast and are often restricted by their training set of known structures. Their scores are often based on similarities to the training set [45] and lack physical meaning, which hampers the interpretation of the results (validity problems). Comprehensive reviews [31,34] and tests [46,47] have shown that the available approaches still have serious limitations with respect to the docking of peptide ligands.

Fragment-based docking is a popular and widely used approach in drug design [48,49,50,51,52], and is based on the linking of docked fragments into the whole bound ligand structure. The number of fragment-based docking methods applied for peptide ligands is still limited. The covalent linking of fragments [48,53] is a critical step in fragment docking, and its success largely depends on the actual steric situation, including the shape-wise matching and the gap between the two docked fragments. Thus, the available methods have multiple limitations, including the lack of full automation, and their dependency on the diversity and selection of linkers and anchoring fragments [54,55,56,57,58,59,60,61]. Inappropriate steric situations of the fragments often necessitate time-consuming follow-up efforts [62] to achieve a new and appropriate covalent bond between two fragments. Further details of the limitations of covalent linking approaches are summarized in Table S2.

In the present study, a new protocol, PepGrow, is introduced and tested for the docking of histone H3 peptide tails to their target reader proteins. PepGrow aims to overcome the limitations of fragment-based docking techniques described above by replacing the fragment linking steps with a growing procedure. Thus, the new protocol is based on the in situ growing of a fragment seed of the peptide ligand in the binding pocket of the reader protein. In drug design, growing steps have been applied for the attachment of small functional groups to ligands [63,64], so as to increase the strength of target–ligand interactions [52]. On the other hand, the growing of a full peptide ligand structure from a small fragment seed is a more difficult task than that handled in the present study. We report the answers to the above challenges, and present a description and validation of PepGrow, comparing its performing with that of of ten other docking methods.

## 2. Results and Discussion

### 2.1. Histone Systems and Benchmark Methods

Ten complexes of histone H3 peptides and reader proteins (Table S1) of physiological importance, a complete N-terminal end, and available apo forms of the reader proteins were collected from the Protein Data Bank (PDB [65]) as test systems for the development and evaluation of PepGrow. Due to problems regarding their structural determination (see Section 1), there are relatively few complexes in the PDB with histone ligands of a complete N-terminal end, that is, starting with the first amino acid. Notably, the use of apo target structures allowed a truly unbiased test, excluding any help of the ligand-bound conformation of the pre-formed target-binding sites that may be present in the holo structures.

**Figure 2:**
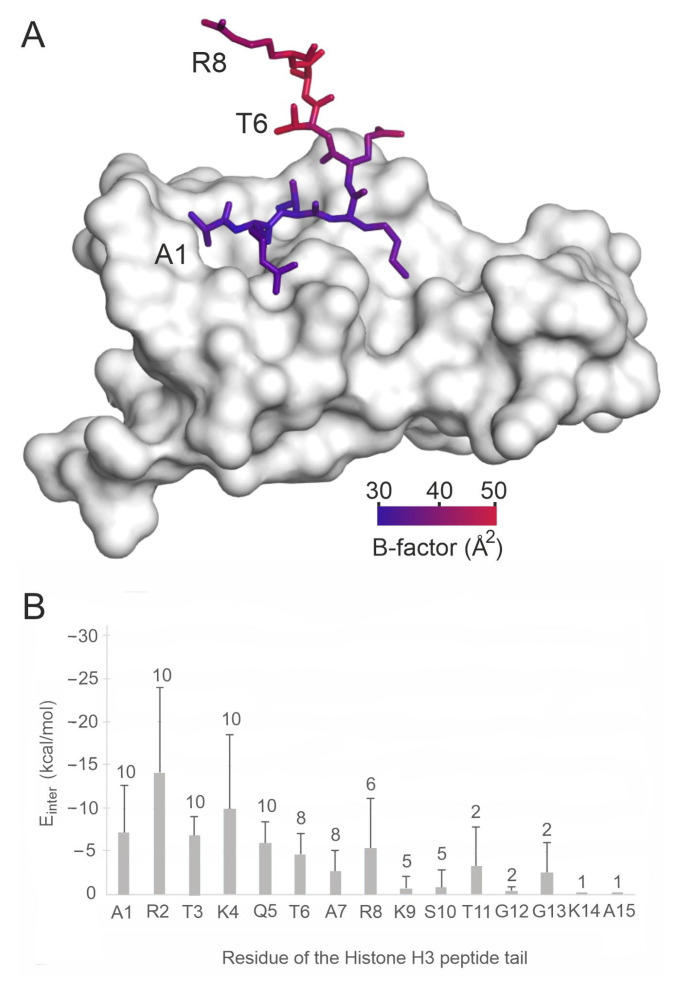
Per-residue energetic and structural analysis of histone H3 peptide ligands bound to their reader proteins. (**A**) The experimental structure of reader UHRF1 PHD finger (grey surface, PDB ID 3sou) in complex with a histone H3 peptide (sticks, colored by Cα B-factors). (**B**) The mean (columns) and standard deviations (error bars) of E_inter_ values for the respective residues calculated for the energy-minimized experimental histone complexes presented in Table S1. The numbers on top of the error bars show the number of systems used for calculation of the averages. The numbers are smaller than the maximum of 10 if histone peptides shorter than 15 amino acids in length were measured experimentally.

Histone H3 peptides contain up to ca. 50 rotatable bonds (Table S1), that is a challenge of computational docking.. The challenges are further increased by the unique binding pattern of histones. Reader proteins often have a shallow binding surface, as in the case of the UHRF1 PHD finger (System 3sou, Figure 2A) [66,67,68]. A considerable part of the linear [69,70] N-terminal region of histone H3 is not able to find anchor points on this shallow target, tending rather to remain unbound in the bulk (Figure 2A). Quantitative analyses of the per-residue interaction energy (E_inter_; see Section 3) distribution of the experimental holo structures in Table S1 show (Figure 2B) that mostly the first five amino acids of the N-terminal of histone H3 are involved in the interaction with the target, while the C-terminal end is exposed to the bulk, and often has a high degree of conformational freedom, which is also reflected by the large atomic B-factors (red in Figure 2A). This finding also emphasizes that only complexes with a full histone tail (i.e., a complete N-terminal end) are useful as test systems.

Besides the PepGrow protocol, a benchmark set of ten available docking methods (Table S3 [41,42,43,44,45,71,72,73,74,75,76,77]) was assembled for the present study. Physico-chemical and hybrid (i.e., incorporating knowledge-based elements into their algorithms; see Section 1) methods were included in the benchmark. The same target and ligand structures were used as inputs for the PepGrow and the benchmark methods.

### 2.2. The PepGrow Protocol

**Figure 3:**
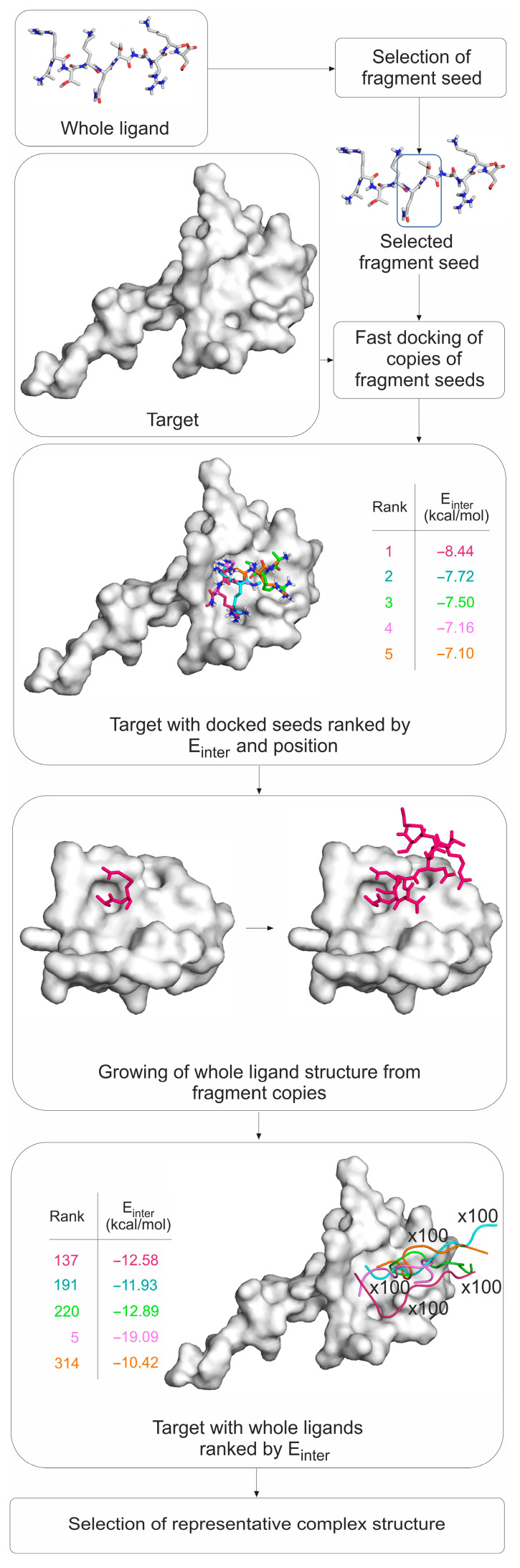
The flow chart of the PepGrow protocol. The different fragment colors correspond to different fragment seed ranks acquired during the fast-docking and seed ranking steps. A close-up of the growth of Rank 1 fragments (purple) only during the growth step is shown for clarity.

**Figure 4:**
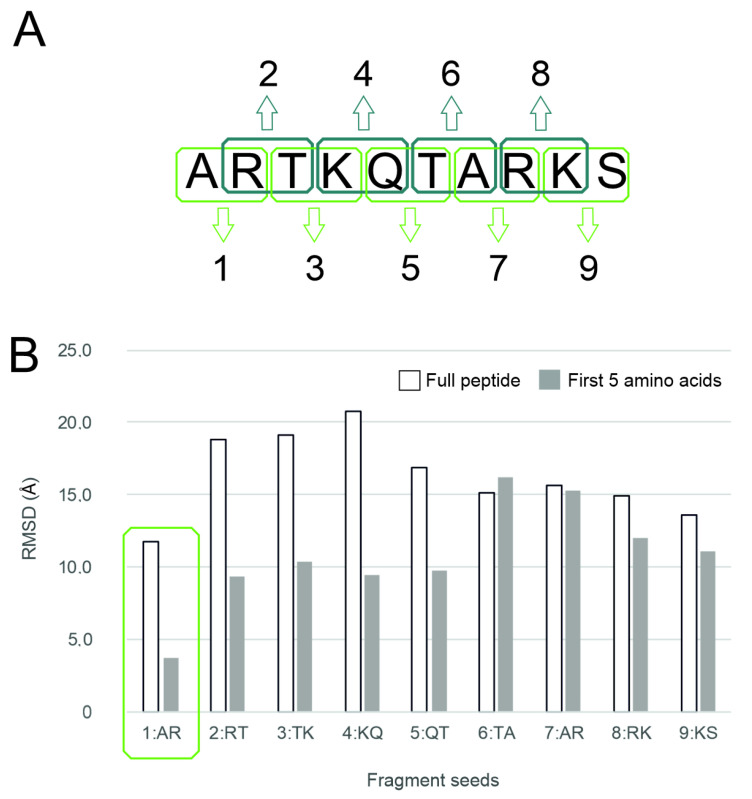
Seed selection. (**A**) All possible (nine) dipeptide fragments were produced from the histone H3 peptide N terminal sequence. Note that Fragment 1 (AR) was capped with an N-methyl group (-NHMe) at the R residue, and Fragment 7 (AR) was capped with an additional acetyl group at the A residue. The capping of the other fragments (2–9) was performed on both ends. (**B**) The PepGrow results for each fragment for System 2ke1. The fragment with the lowest RMSD_top_ is marked with a green frame. See Table S4 for details.

The PepGrow protocol builds the structures of target–peptide complexes at atomic resolutions (Figure 3) without prior knowledge of the binding site residues of the target. PepGrow starts with the selection of a seed molecule that is a fragment of the ligand peptide. As the ligand used in our cases is the same histone H3 tail (Table S1), the selection of an appropriate seed needs to be carried out only once. For the seed selection procedure, the use of only one holo complex structure (2ke1) proved to be sufficient to pick the best dipeptide fragment from among all of the possible dipeptides (Figure 4A) derived from the H3 peptide (Table S4). In the case of histone H3, Fragment 1 (AR) produced the best results (Figure 4B), and therefore, it was selected as the seed for H3 peptide docking for all complexes except for System 2fuu, for which Fragment 4 (KQ) was used. The selection of Fragments 1 (AR) and 4 (KQ) as seeds is also reflected by the per-residue E_inter_ plot (Figure 2B), where R2 and K4 have the largest E_inter_ contribution among the residues of histone H3. (Thus, the fast, per-residue E_inter_ scoring (Figure 2B) plot of a single strong complex is also applicable for seed selection in PepGrow).

In the next step, the seed was docked on the target protein using a fast method utilizing AutoDock 4.2.6, focusing on the peptide binding area [78], which resulted in several binding modes (where the binding mode refers to the position, orientation, and conformation of a ligand). The binding modes were ranked according to the calculated free energy of their binding and their structural similarity. The representative binding modes were produced for all ranks (see Tables S5 and S6 for a list of the rank counts of all systems). All representative binding modes then proceeded to the fragment growing step, which was accomplished using the builder routine of the homology modeling program Modeller [79]. The experimental target structure with the docked peptide fragment (seed) served as a starting template for growing fragments in the binding pocket. In this way, all docking ranks were used to generate thousands of target–peptide complex models in a matter of minutes, resulting in a large enough pool of peptide binding modes (see Tables S5 and S6 for a list of the binding mode counts of all systems). The complex models of the pool were scored and ranked based on the target–ligand intermolecular interaction energy (E_inter_, Section 3) values calculated for the full peptide and for the five N-terminal amino acid residues, respectively. The representative peptide structure with coordinates closest to the average coordinates calculated for the peptide structures ranked in the top 1% (Rank 1) according to E_inter_ was selected as the solution. It was observed that in many cases, the top 1% of solution structures contained the best one, but not necessarily the best E_inter_ of all of the structures. Thus, it was reasonable to consider a structure that was representative of the top 1%, rather than a single top structure. Technical details of the PepGrow protocol are provided in the Section 3. Example in- and output files and computational details of the PepGrow protocol are available in the Public Repository files Protocol.pdf and Protocol.tgz (see Data Availability Statement).

### 2.3. Performance

**Figure 5:**
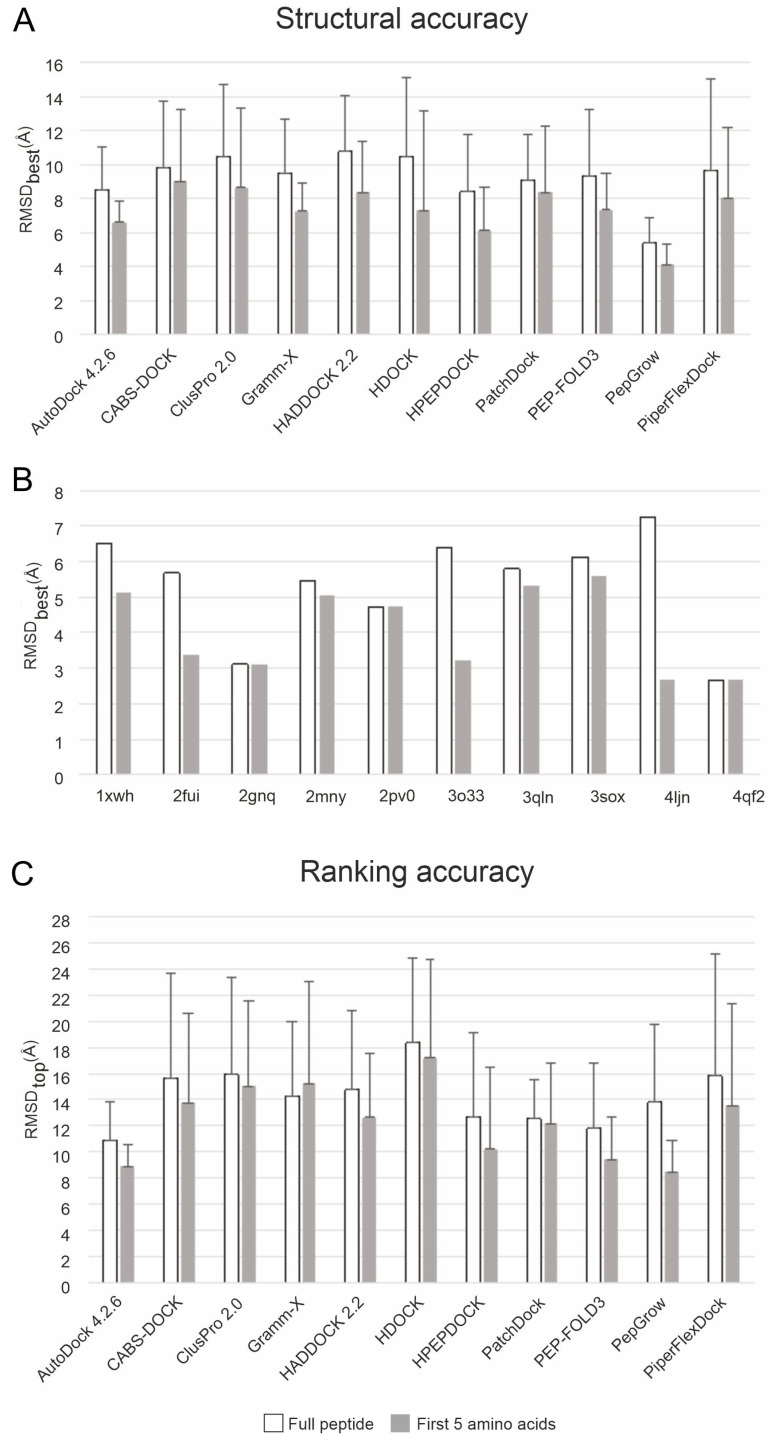
The statistics of docking results obtained for all test systems of Table S1 using all apo target structures. (**A**) Columns represent the mean RMSD_best_ values (of all test systems) calculated for ligand-binding modes supplied by PepGrow and the 10 benchmark methods. Error bars represent standard deviations (see also Table S7a). (**B**) Structural performance of PepGrow on the individual test systems (see also Table S5). (**C**) Columns represent the mean RMSD_top_ values (of all test systems) calculated for ligand-binding modes supplied by PepGrow and the 10 benchmark methods. Error bars represent standard deviations (Table S7a).

The structural accuracies of PepGrow and 10 other docking methods are expressed as the root mean square deviation (RMSD; see Section 3) measured between the docked and the experimental (reference) ligand-binding modes. As the experimental complexes mostly show stable (reference) conformations at the first five amino acids of histone H3 (Figure 2), RMSD values were calculated for the full ligand and for the first five amino acids of the N-terminal, respectively. The lowest RMSD of all docked binding modes is referred to as RMSD_best_. The statistics (mean and standard deviation) for the RMSD_best_ values of docking results to the apo targets for all systems in Table S1 are presented in Figure 5. Due to the high mobility (and structural uncertainty) of peptide ligands outside the binding interface, it is common to use only the interfacial (strongly bound core) amino acids [75] for RMSD calculation. In the case of the histone H3 ligand, this core region corresponds to (see Section 2.1) the first five amino acids (full bars in Figure 5). For comparison, the RMSD values measured for all amino acids (empty bars in Figure 5) of the docked histone H3 ligands are also shown. In general, the RMSD_best_ values calculated for the first five amino acids of H3 reflect a much better performance for all methods than the RMSD_best_ values calculated for the full ligand (Figure 5A), due to the natural flexibility of the extended C-terminal region described above (Figure 2).

**Figure 6:**
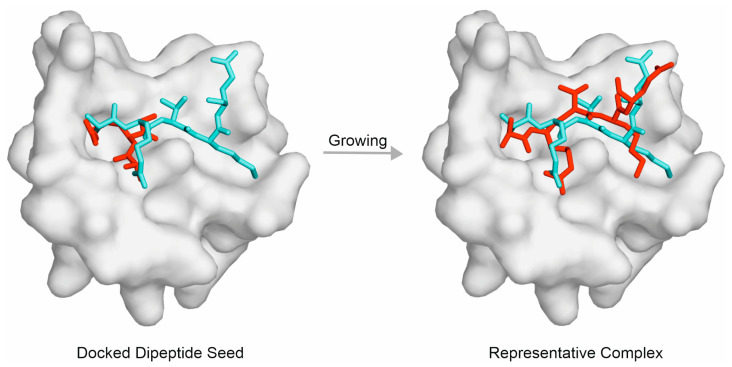
Fragment growing of the fast-docked seed for the complex of the human BAZ2A PHD zinc finger reader (grey surface)–histone H3 peptide (sticks, System 4qf2). The fast-docked seed AR-NHMe of an RMSD of 3.79 Å is shown as red sticks (**left**), representing a good basis of peptide growing. The ligand structure corresponding to an RMSD_best_ of 2.67 Å is shown as red sticks (**right**), representing the results of the growing. The crystallographic ligand-binding mode is shown as teal sticks for comparison.

The statistics regarding the apo targets show that PepGrow outperformed all of the other fast docking approaches (Figure 5A), with an RMSD_best_ of 5.36 (±1.47) Å being calculated for all of the amino acids of the docked histone H3 peptide fragments. Furthermore, PepGrow achieved an excellent RMSD_best_ of 4.09 (±1.18) Å, when calculated for the first five amino acids, as well. The per-system analysis of the PepGrow results (Figure 5B) indicates that the best performance was obtained in the case of the target human BAZ2A PHD zinc finger (System 4qf2). Here, the AR-NHMe dipeptide seed was accurately docked (Figure 6), providing a good starting point for ligand growing. The docking of such dipeptides can be accomplished precisely [80] using fast docking techniques. Thus, they provide a good starting point for growing peptide ligands, which is a better alternative than the problematic linking of several, often inadequately docked large-peptide fragments. The accurately docked dipeptide seeds also have the best E_inter_ values (Figure 2), determining the success of PepGrow.

Target flexibility poses a great challenge for docking methods [81]. To check the sensitivity of the investigated docking methods to target conformation, all docking calculations were repeated for the holo structures of the target molecules. As the holo structures have a pre-formed conformation that is ideal for binding to a certain ligand, large differences between the results when docking to the apo and when docking to the holo forms may indicate a high (unwanted) sensitivity to target conformation and moderate robustness of the method. In the case of PepGrow, no significant differences could be detected (Figure 5 vs. Figure S1) between the results on the apo and holo targets, indicating the robustness of the method.

The acceptable level of RMSD_best_ was concluded to be 4.0 ± 3.0 Å on the basis of data (Table S8) collected from publications related to the benchmark methods (Table S3), in which RMSD was calculated only for the peptide backbone. Notably, side-chain atoms were also included in the RMSD calculations in the present study. Thus, the above performance of PepGrow can be considered to be as good as or above average when compared to the RMSD values produced by the benchmark methods (Figure 5).

Besides the structural accuracy of the methods, their ranking performance was also measured on the basis of their respective RMSD values. The docked-ligand-binding modes were ranked by the default scoring functions of the respective methods (Table S3, Supplementary Materials). The RMSD value of the ligand with the best score (representative of the first rank) is referred to as RMSD_top_. In the case of a method with perfect scoring and ranking, RMSD_top_ is equal to RMSD_best_ per definitionem. Unfortunately, such an ideal situation was not observed with the methods investigated, as RMSD_top_ considerably exceeded RMSD_best_ in all dockings to the apo targets (Figure 5C), and the same trend was observed in the cases of holo forms (Figure S1). A comparison of the ranking performance of all of the methods (Figure 5C) shows that PepGrow achieved the best results when compared to the benchmark methods. Thus, the E_inter_-based representative selection method of PepGrow is a viable ranking alternative. Notably, the separate components of E_inter_ (Lennard-Jones and Coulomb terms, respectively) showed a drop in performance (Table S9), and therefore, E_inter_ including both terms (see Section 3) was used in the ranking throughout the present study.

The above results indicate that the structural (Figure 5A) and ranking (Figure 5C) performances of PepGrow are better than/comparable to those of the 10 benchmark methods presented in Table S3. PepGrow can also be considered a physico-chemical method, with energy-based scoring and ranking of the ligand-binding mode (Section 2). In theory, physico-chemical methods are generally applicable for any ligand type with appropriate molecular mechanics parametrization. The efficient sampling of the conformational space of flexible peptide ligands [82] like histone H3 tails is a common problem for all fast docking methods. Knowledge-based and hybrid methods (Table S3) attempt to solve this problem using a training set of experimentally determined structures as templates for achieving the correct bound ligand conformation. However, their performance is limited by the availability and reliability of templates for use in training.

In addition to the above sampling problem, the scoring functions of fast docking methods (Table S3) tend to maximize the interactions of the entire ligand with the target, and therefore cannot handle non-interacting parts (see Section 1). Fragment docking methods may provide a solution for this scoring problem by docking only short fragments instead of the entire ligand. This may be a divide-and-conquer strategy for addressing the limitation of linking fragments (see Section 1). For example, PIPER-FlexPepDock is a fragment-based, hybrid approach in which an ensemble of short peptide fragments is collected from experimentally determined structures with a high degree of sequence and (predicted) secondary structure similarity to the actual ligand. However, such methods are also limited by the lack of structures of peptide fragments of large size and/or unusual conformations. Similar to PIPER-FlexPepDock, PepGrow utilizes the potential of physico-chemical methods to accurately dock small peptide fragments, but instead of all possible fragments in the peptide, it focuses on the anchoring fragment of a good E_inter_ (see Section 2, Figure 2) and grows the remaining part of the peptide in situ in the binding pocket. Thus, PepGrow addresses both the sampling and scoring (ranking) problems via its fragment docking strategy and the focused growing of a ligand from the docked seed the strongest interaction with the target.

Data files of the performance tests of PepGrow and the benchmark methods are available in the Public Repository files PepGrow.tgz and Benchmark.tgz (see Data Availability Statement).

## 3. Materials and Methods

### 3.1. Selection of Test Systems and Benchmark Methods

All atomic coordinates of the targets were acquired from the PDB. Apart from their physiological relevance, histone-target systems were preferentially selected that exhibited high resolution (<4 Å) and the availability of a non-covalently bound histone H3 N-terminal peptide tail, starting from the first amino acid (A). The availability of both complexed (holo) and apo forms was a selection criterion, as well. For the benchmark methods, fast docking engines were selected that were designed to model interactions in protein–peptide or macromolecular complexes (except AutoDock) and had previously been evaluated on protein–peptide complexes. A further selection criterion was their free availability for academic purposes via web servers or as standalone programs. The investigated docking engines can be roughly sorted into knowledge-based, physico-chemical, and hybrid categories (Table S3).

### 3.2. Performance Metrics

Both structural and ranking performance are expressed in terms of root mean square deviation (RMSD), a commonly used measure for the comparison of the conformational match of two molecules. In the present study, the bound conformation of a peptide ligand produced by PepGrow (P) was compared to the bound conformation of the same ligand in the experimental complex (E) structure used as a reference (Equation (1)).

(1) $$RMSD=\sqrt{\frac{1}{N}\sum_{n=1}^{N}{\left|P_{n}-E_{n}\right|}^{2}}$$

*N* is the number of ligand heavy atoms, ***E*** is the space vector of the nth heavy atom of the experimental reference ligand molecule, and ***P*** is the space vector of the nth heavy atom of the PepGrow-calculated ligand conformation. Crystallographic structures were mostly used as references (Table S1). In 3 cases, NMR structures were also employed, where the first model was selected as a reference. RMSD values were calculated after superimposition of the target parts (Table S10).

### 3.3. Application of Benchmark Methods

The general and specific settings, and the preparation of targets and ligands are detailed for all benchmark methods in the Supplementary Materials Methods [83,84,85,86,87,88,89].

### 3.4. PepGrow

Target preparation. The atomic coordinate structure files for the selected target protein (Table S1) were downloaded from the PDB. All non-protein parts (ligands, waters, etc.) were removed from all selected target structures prior to docking. If the structure was a homo-oligomer, then only one selected chain was used (the first protein chain in the PDB file). The rest of the target molecule was equipped with polar hydrogen atoms and Gasteiger–Marsilli [90] partial charges in AutoDock Tools [44].

Ligand preparation. An initial fragmenting step was used to create dipeptide-sized fragments of the original histone H3 peptide. The fragments were built using the Tinker program package [91] with the protein, newton and xyzpdb commands. The cut was made between the carbon and nitrogen atoms of the amide bond, acetyl (Ac-) and N-methyl (-NHMe) groups were used to block the N- and C-terminal cut ends (the 1:AR fragment was not capped at the N-terminal end, but the 7:AR fragment was capped at both ends). These blocking groups were added in Tinker [91]. The acquired ligand structures were then energy minimized using Open Babel [92] with the Amber99 force field [93] using the steepest descent optimization with 10^4^ steps; the convergence threshold was set to 10^3^ kJ mol^−1^ nm^−1^. The next step was conjugate gradient minimization; a maximum of 10^4^ steps was used, and the convergence threshold was set to 10 kJ mol^−1^ nm^−1^. Gasteiger–Marsili charges [90] were added to the fragments with AutoDock Tools [44]

Fragment docking. The fragment docking was performed using AutoDock 4.2.6 [44]. The previously prepared target was handled as a rigid body. All active torsions were allowed on the prepared ligand fragments. All ligand structures were docked to the interacting site defined by the experimental structure, where the docking box was set to a size that would fit the whole peptide inside. The number of grid points was set to 60 × 60 × 60, with a grid spacing of 0.375 Å; the middle of the box was set to the center of the respective experimental full ligand conformation in a manner similar to the procedure used for the benchmark methods. The Lamarckian genetic algorithm was used to perform a global search. Ten docking runs were performed, and the resulting fragment conformations were ranked [94], and representatives of each rank were used in Step 4.

Fragment growing with a homology modelling tool. All docked fragment copies were processed using Modeller 9.22 [79], a homology modeling program. The template structure was the experimental structure of the target protein with the docked (previous step) fragment seed of the ligand peptide. The query sequence was the respective sequence of each system and the histone H3 peptide tail matching the sequence length seen in the corresponding experimental structures (Table S1). The target and ligand sequences were taken from the UniProt database. The alignment between the template structure and the query sequence was manually optimized if necessary to obtain identical regions that correctly matched each other. This was necessary when fitting the sequence of the docked dipeptide seed to the sequence of the whole ligand. The Modeller 9.22 software package was applied to generate 100 models per step, following the final PepGrow protocol. Explicit manual restraints were not added to access additional energy calculation features. During the method development phase of the present work, restraints, energy calculating features, and seed number variation steps were evaluated thoroughly (Table S11). When the rapid generation of 100 models with default building settings was compared with the generation of fewer models (20) with slower refinement, the results were similar, so the faster method (with 100 models) was selected as the main PepGrow protocol step. The robustness of the building procedure was further challenged by changing the random seed number, which did not affect the results (Table S11). For System 2fuu, fragment 4:KQ was selected, due to the special interaction of the trimethylated K4 with the target. In addition, fragment 4:KQ had the second-best performance (after 1:AR) when compared with the other seeds (Figure 4).

Scoring. To extend the use of the method to apo structures with previously unknown N-terminal histone tail ligand positions, it is important to apply a scoring function that is able to select the bound ligand conformation closest to the real structure. The discrete optimized protein energy (DOPE [79,95]), the Modeller probability density function (molpdf [79,95]), and the Lennard-Jones, Coulomb and E_inter_ interaction energy scores (Equation (2)) of each model were calculated. The E_inter_ interaction energy score calculated for the first five amino acids was the basis of the representative model selection (Tables S7a,b and S12). Table S13 details the scoring functions of the benchmark methods; the differences between the physico-chemical, knowledge-based and hybrid methods were determined based on these scoring functions. Notably, the DOPE and molpdf scores were developed on a benchmark set containing only single-chain proteins, according to the User’s Manual of Modeller 9.22 [79]; there is no guarantee of their applicability to multi-chain structures. The calculated DOPE and molpdf scores were therefore only used to test the effect of changing the random seed number for model generation during the initial steps of testing Modeller, as these two scores are the default scoring functions of the software (Table S11).

### 3.5. Calculation of E_inter_ and Energy Analyses 

Experimental, Modeller-built, and energy-minimized experimental structures were subjected to per-residue interaction energy scoring. The missing atoms of all crystallographic targets were modeled using SWISS-Model [96]; for a detailed list of the missing atoms and residues, please see the respective pdb structure files. However, these missing atoms did not affect the binding site. The experimental structures were equipped with polar hydrogen atoms and Gasteiger–Marsilli partial charges [90] using Open Babel 2.4.0 [92], and were converted from pdb files to mol2 files. The mol2 files were then subjected to per-residue interaction energy calculation using Equation (2), implemented in an energy calculator program, which is available as a binary version, downloadable as PepGrow.tgz (see Data Availability Statement). Lennard-Jones and Coulomb energies were calculated and summarized to obtain the total E_inter_ for each residue, and the whole ligand according to Equation (2). The Coulomb term was calculated with a distance-dependent dielectric function of Mehler and Solmajer [97] (Equation (3)), and Amber 2012 van der Waals parameters and atom types were used [98].

(2) $$\begin{array}{c} E_{inter}=E_{LJ}+E_{Coulomb}=\sum_{i,j}^{N_{T}N_{L}}\left(\frac{A_{ij}}{r_{ij}^{12}}-\frac{B_{ij}}{r_{ij}^{6}}+\frac{q_{i}q_{j}}{4\pi \varepsilon_{0}\varepsilon_{r}r_{ij}}\right)\\ A_{ij}=\varepsilon_{ij}R_{ij}^{12}\\ B_{ij}=2\varepsilon_{ij}R_{ij}^{6}\\ R_{ij}=R_{i}+R_{j}\\ \varepsilon_{ij}=\sqrt{\varepsilon_{i}\varepsilon_{j}} \end{array}$$

where ε_ij_ is the potential well depth at equilibrium between the ith (ligand) and jth (target) atoms; ε_0_ is the permittivity of vacuum; ε_r_ is the distance-dependent relative permittivity (Equation (3)); R_ij_ is the inter-nuclear distance at equilibrium between the ith (ligand) and jth (target) atoms; q is the partial charge of an atom; r_ij_ is the actual distance between the ith (ligand) and jth (target) atoms; N_T_ is the number of target atoms; N_L_ is the number of ligand atoms.

(3) $$\varepsilon_{r}=A+\frac{B}{1+ke^{-\lambda Br}}$$

where B = ε_0_ − A, ε_0_ is the dielectric constant of water at 25 °C, and A, λ and k are constants [97].

## 4. Conclusions

Although fast docking methods have proven successful in the design of small-molecule ligands [99,100], they face persistent challenges [99,100,101,102]. While long peptides are often used as templates for the development of new drugs [103,104,105], they are especially challenging ligands due to their high degree of flexibility and hydration, which cannot properly be handled by fast docking methods. In the present study, a popular fast docking method, AutoDock 4.2.6, and the fast model building function of the widely used program Modeller were combined into a new protocol PepGrow.

A comparison of the results with those obtained using ten other benchmark methods showed that PepGrow offers a real alternative for the construction of histone complexes. The relatively good performance of PepGrow is based on at least two key components of the algorithm. Firstly, the docking of very short and strongly interacting (di)peptide seeds can be reliably achieved [80] using currently available fast docking methods like AutoDock 4.2.6 (unlike large peptide ligands, where fast docking presents problems [31]). Secondly, instead of the problematic linking step of all fragments of the ligand, a robust ligand growing step is implemented.

PepGrow constructs the complex structures of histone H3 peptides of various lengths with various targets. While the number of such complexes is expected to be very high (histone code), only a small number of structures have been determined. Thus, PepGrow can help to accelerate the structural exploration of the histone code, as well as the prediction of the outcome of the reader–DNA binding competition mentioned in the Introduction. The disordered nature of histone peptides presented a real challenge for all eleven methods compared. The structural performance of PepGrow was better than that of the other methods, the ranking of such large ligands still remains [34,37] a challenging task for all methods. Our results also indicate that physico-chemical scores like E_inter_ are a necessary component of the ranking and selection of representative structures. The histone complexes selected for the present work can be recommended as a particularly challenging test set for future method development studies.

## Data Availability

All data files are available at https://zenodo.org/record/8251745 (access date: 20 August 2023). A compressed data file PepGrow.tgz contains the in- and output files of PepGrow for both the holo and apo systems, the scripts and the programs necessary to produce them, and a Table of Contents file with detailed folder descriptions. A portable document file Protocol.pdf contains detailed description how to perform the PepGrow protocol. A compressed data file Protocol.tgz contains example folders of PepGrow on a system with in- and output files, scripts and the programs necessary to produce them, and a Table of Contents file with detailed folder descriptions. A compressed data file Benchmark.tgz contains the in- and output files of the 10 benchmark methods for both holo and apo systems, and a Table of Contents file with detailed folder descriptions.

## Supplementary Materials

The following supporting information can be downloaded at: https://www.mdpi.com/article/10.3390/ijms241813831/s1.
