# Supplementary material for: Construction of Histone–Protein Complex Structures by Peptide Growing
Source: Int J Mol Sci. 2023 Sep 7;24(18):13831. doi: 10.3390/ijms241813831 (PMC10530865; doi:10.3390/ijms241813831)
Supplement: Supplementary file 1 [file ijms-24-13831-s001.zip › ijms-2595908-supplementary.pdf]

Supplementary Material

Construction of histone-protein complex structures by peptide growing

Balázs Zoltán Zsidó[†], Bayartsetseg Bayarsaikhan[†], Rita Börzsei, Csaba Hetényi*

Pharmacoinformatics Unit, Department of Pharmacology and Pharmacotherapy, Medical School, University of Pécs, Szigeti Út 12, 7624 Pécs, Hungary

[†]Equal contribution.

*Corresponding author.

Table of Contents

Table S1 2
Table S2 4
Table S3 8
Table S4 10
Table S5 11
Table S6 12
Table S7 13
Table S8 15
Table S9 16
Table S10 17
Table S11 18
Table S12 19
Table S13 20
Figure S1 24
Supplementary Methods 25
References 29

Tables

Table S1 Test systems

Holo structure (PDB code)	Resolution (Å)	Apo structure (PDB code)	Resolution (Å)	Target	Peptide		(Patho)physiological importance	K_d (μM)
					sequence[1]	#NRB[3]		
4q6f	1.91	4qf2	1.7	BAZ2A PHD Zinc finger	ARTKQ	25	Prostate cancer (Bortoluzzi et al., 2017)	2.51 (ITC, [1], 9 amino acids)
2co0	2.25	2gnq	1.8	WDR5	ARTKQTARKSTGGKA	25	Hematopoiesis, vertebrate development (Alexander J Ruthenburg, Wooikoon Wang, Daina M Graybosch, Haitao Li and Allis, Dinshaw J Patel, 2006)	3.3 (SPR, [2], 14 residues)
2pvc	3.69	2pv0	3.3	DNMT3L	ARTKQTA	32	Meiotic defects in spermatocytes, heritable transcriptional silencing (Ooi et al., 2007)	2.1 (Fluorescent polarization,[3])
3qlc	2.5	3qln	1.901	ARTX ADD	ARTKQTARKSTGGKA	32	Mental retardation, α-Thalassemia (Iwase et al., 2011)	3.7 (ITC, [4], 21 amino acids)
3sou	1.8001	3sox	2.6501	UHRF1 PHD finger	ARTKQTARK	39	Colorectal carcinoma (Houliston et al., 2017; Rajakumara et al., 2011)	2.1 (ITC, [5], 10 amino acids)
3o37	2.0	3o33	2.0	TRIM24 PHD-Bromo complex	ARTKQTARKS	50	Breast cancer, hepatocellular carcinoma (Tsai et al., 2010)	8.6 (ITC, [6], 15 amino acids)
2ke1	Solution NMR[2]	1xwh	Solution NMR	AIRE PHD finger	ARTKQTARKS	50	Autoimmune polyendocrinopathy-candidiasis-ectodermal dystrophy (Org et al., 2008)	5.3 (FRET,[7]), 6.5 (ITC, [8])
2mnz	Solution NMR[2]	2mny	Solution NMR	KDM5B PHD1 finger	ARTKQTARKS	50	Breast cancer cell proliferation (Zhang et al., 2014)	6.4 (ITC, [9], 21 amino acids)
4lk9	1.6	4ljn	3	MOZ double PHD finger	ARTKQTARKSTGGKAPRKQLA	60	Intellectual disability, leukemia (Klein et al., 2020)	-
2fuu	Solution NMR[2]	2fui	Solution NMR	BPTF PHD finger	ARTKme3QTARKSTGGKA	70	Breast cancer (Li et al., 2006)	2.7 (ITC, [10], 15 residues)

[1]The **underlined parts** of the amino acid sequences of the histone tails are representing the amino acids **captured by the indicated experimental method**, and the non-underlined parts were in the solution or crystal (depending on the method), but not in the coordinate file.
[2]For NMR complex structures, ligand structures from their first model were used as reference structures.
[3]The number of rotatable bonds (NRB) is calculated for the underlined sequence of the peptide.

Table S2 Approaches and limitations of linking of docked fragments

Program name	Biomolecules (ligand-target)	Description of the linking method	Limitations of linking
Chauvot de Beauchene et al. [11]	ssRNA-protein	RNA sequence is cut in overlapping trinucleotides, and their ensembles of conformers are docked by ATTRACT, starting from the fragments with anchoring nucleotide(s) with position restraints determined by the docking results of their adjacent fragments.<br>The top-scored poses of adjacent fragments with RMSD less than 1.4 Å between their overlapping nucleotides are arranged into chains of spatially overlapping poses.<br>From overlapping fragments in the chain, nucleotides closest to the anchoring nucleotide(s) are kept in the nucleotide chain.<br>Nucleotides in the chains are disconnected and in a coarse-grained presentation which was converted into all-atom representation by their mono-nucleotide library based on the RMSD after fitting. The nucleotides were connected and clashes were solved using energy minimization.	Not fully automated usage of coarse-grained representation<br>Dependency on the number of anchoring fragments<br>High computational cost
Liao et al. [12]	peptide-protein	Peptides were split into two fragments and docked separately by AutoDock Vina.<br>To merge the fragments, the last two residues of fragment 1 and the first two residues of fragment 2 are fitted into and replaced by those residues of 100 ns-long MD runs.<br>Rebuilt peptides are locally optimized and scored by AutoDock Vina, top 1000 structures are selected.<br>Selected structures further refined by MD simulations	Not fully automated<br>High computational cost

Fragment-based flexible ligand docking Budin et al. [13]	small molecule-protein	the SEED program docks a library of functional groups to the target.<br>Three anchoring fragments of the ligand are chosen and a functional group with the highest similarity is selected for each fragment.<br>The full ligand is docked into the binding site determined by the geometric centers of top-ranked poses of the corresponding functional groups of the three selected fragments.<br>The anchoring fragments are only used to determine positional restraints during docking.	Not automatized<br>Not applicable for high-throughput screening
eHiTS protocol [14]	small molecule-protein	The ligand is divided into rigid and flexible fragments, during fragmentation.<br>Both atoms of the fragmented bond are duplicated (join atoms).<br>Docking rigid fragments using shape complementary method.<br>Combinations of docked poses of rigid fragments where distances between them are compatible with the flexible fragment are produced using a hyper-graph clique detection algorithm.<br>Flexible fragments are fitted into each set of rigid fragments by selecting the lowest energy conformers with ending atom pair distances similar to those required and followed by a local minimization.<br>Rigid and flexible fragments are connected through overlay of the joint atoms.<br>Further energy minimization.	Docked fragments move during energy Minimization step of linking
CONFIRM [15]	small molecule-protein	Bridge connectors for a set of fragment molecules are searched from a pre-prepared library based on the distances between the attachment points and their atom types.<br>The resulting connectors are attached to the fragments by overlaying the bridge termini to	Dependency on the Diversity of pre-prepared bridge libraries

		the anchors on the fragments using PilotScript.<br>Connected ligands are then docked by Glide 4.5.<br>Energy minimization	
Samsonov et al. [16]	Glycosaminoglycans-protein	Glycosaminoglycan (GAG) ligands were divided into fragments of possible dimers, trimers of monosaccharides and docked by AutoDock 3.<br>Docked poses are converted into coarse-grained representation and connected to each other based on RMSD between their overlapping parts.<br>The assembled chains are converted into chains of monomers by averaging the atomic positions of the overlapping parts of the poses. Calculated average coordinates of each monomer were replaced by the best-fitting monomer in the library in all-atom representation. The monomer library for each GAG residue consists of docking results of the monomer for all complexes in the test set.<br>Energy minimization.	Usage of coarse-grained representation
Fragment Blind Docking [17]	protein-peptide	Pairwise linking of fragments, probing all possible pairwise combinations and producing the longest possible peptide. The linking is based on a distance matrix between C_T and N_T atoms of the fragments, the user can define distance tolerance for the aforementioned distance. To re-form the covalent bond welding is performed, which includes rotation of the fragments. After welding, an energy minimization is performed with explicit water molecules to yield the final structure.	The fragments are rotated in order to achieve correct positions for re-forming the covalent bonds.<br>Not automatized.
FlexX [18]	small molecule-protein	The anchoring fragment is user determined, the remaining part of the ligand is cut at each rotatable bond.	Dependency on the selection and docking accuracy of the anchoring fragment

		The anchoring fragment is docked by RigFit, a rigid-body docking method.<br>The new fragment is added to each pose of the previous fragment in all possible conformations without having clashes.<br>Fragments are linked by their compatibility to a torsional database.<br>For each extended fragment, new interactions with the receptor are searched.<br>Optimization of extended fragments using weighted superposition of points.<br>The best-extended fragments are selected and used for the next iteration.	Assumes that the base fragment interacts with the active site.

Table S3 Benchmark methods tested in the present study

Method name	Search method	Scoring	Ranking/Clustering	Ref
**Physico-chemical methods**				
AutoDock 4.2.6	LGA[1], GA[2], simulated annealing	Weighted sum of Lennard-Jones 6/12 potential, H[3]-bonding term, Coulombic electrostatic potential, desolvation, pairwise atomic term	Binding energy-based ranking and conformational similarity-based clustering	[19]
CABS-dock	Generation of random conformations of the peptide followed by Replica Exchange MC[4] dynamics, reconstruction of final models using Modeller with DOPE[5] statistical potential	Weighted sum of short range sequence-dependent and independent interaction terms, H-bonding term, repulsive interactions term, and long range pairwise interactions term	Binding energy-based ranking and k-medoids clustering	[20]
ClusPro 2.0	FFT[6]-based PIPER docking followed by energy minimization of selected models	Weighted sum of shape complementary term, electrostatic energy term and DARS[7] potential	Binding energy-based ranking and conformational similarity-based clustering	[21]
GRAMM-X	FFT-based rigid-body docking with smoothened potentials	Sum of Lennard–Jones potential, pair potentials, cluster occupancy, degree of the evolutionary conservation of the predicted interface	Binding energy-based ranking	[22]
HADDOCK 2.2	Generation of random conformations of the peptide followed by AIRs[8] guided rigid-body docking, three simulated annealing refinements and refinement in explicit solvent	Weighted sum of van der Waals, electrostatic terms, desolvation term and AIR energy term with the buried surface area	iRMSD[9]-based clustering and ranking based on average binding energy and average buried area	[23]
PatchDock	Shape complementarity-based Geometric Hashing method	Sum of geometric fit and atomic desolvation energy	Score-based ranking and RMSD-based clustering	[24]
PEP-FOLD3	Generation of peptide conformations where geometric descriptors are described by states of a Hidden Markov Model and the probabilities of each fragment of the peptide to have each state is calculated by Support Vector machine, followed by Taboo sampling algorithm, and MC-based refinement	sOPEP force field: local energy terms (bond lengths, angles, improper torsions and torsional angles), non-bonded terms (van der Waals terms), and H bonding term	RMSD-based clustering without superimposition and binding energy-based ranking	[25]
**Hybrid methods**				
HPEPDOCK	Generation of peptide conformations with MODPEP program, followed by ensemble docking with MDock program and energy minimization with SIMPLEX algorithm	ITScore, an iterative knowledge-based scoring function	Binding energy-based ranking	[26]
HDOCK	Template-based model building for target and ligand, MD refinement of models, followed by separating ligand and target structures from the models and docking with FFT-based HDOCKlite docking program	ITScorePP, an iteratively derived knowledge-based scoring function	Score-based ranking and RMSD-based clustering	[27]
PIPER-FlexPepDock	Peptide fragment extraction from experimentally determined structures based on sequence and secondary structure similarity by Rosetta fragment picker and docking by FFT-based PIPER program followed by MC-based refinement with Rosetta FlexPepDock	Weighted sum of shape complementary term, electrostatic term, decoys as the reference state pairwise term	RMSD-based clustering using a hierarchical clustering algorithm and ranking based on the binding energy of their representatives	[28]

[1]LGA, Lamarckian Genetic Algorithm
[2]GA, Genetic Algorithm
[3]H, hydrogen
[4]MC, Monte-Carlo
[5]DOPE, Discreet Optimized Potential Energy
[6]FFT, Fast Fourier Transform
[7] DARS, Decoys as the Reference State
[8]AIRs, Ambiguous Interaction Restraints
[9]iRMSD is the backbone root-mean-square displacement of the ligand residues of the interface in the modelled versus the experimental target structures calculated after the targets of these structures have been superimposed.

Table S4 The fragment seed selection based on docking and homology modelling steps performed on the holo 2ke1 system.

	$RMSD_{top}$	$RMSD_{top,5}$ [a]	$RMSD_{best}$	$RMSD_{best,5}$ [b]
1:AR	11.79	3.66	6.43	3.66
2:RT	18.81	9.37	5.88	3.16
3:TK	19.12	10.35	6.86	3.03
4:KQ	20.73	9.47	10.52	6.81
5:QT	16.91	9.73	10.59	6.89
6:TA	15.12	16.20	12.45	8.25
7:AR	15.59	15.31	9.59	7.12
8:RK	14.94	12.03	9.57	7.29
9:KS	13.61	11.07	10.87	8.91

[a] $RMSD_{top,5}$ is the top ranked structure based on the respective interaction energy calculated only for the first 5 amino acids.

[b] $RMSD_{best,5}$ is the best ranked structure based on RMSD calculated only for the first 5 amino acids.

Table S5 Average of best 1% models for PepGrow results with apo proteins when ranked according to their $RMSD_{full}$ and $RMSD_5$

	**$RMSD_{full}$**		**$RMSD_5$**			
PDB ID	**Mean (Å)**	**SD (Å)**	**Mean (Å)**	**SD (Å)**	**# model***	**Docking ranks**
1xwh	6.87	0.35	5.29	0.16	300	3
2fui	6.05	0.31	3.54	0.09	500	5
2gnq	3.92	0.63	3.92	0.63	500	5
2mny	5.86	0.40	5.16	0.07	400	4
2pv0	5.87	0.97	5.15	0.27	500	5
3o33	6.48	0.09	3.62	0.40	700	7
3qln	5.94	0.10	5.45	0.13	400	4
3sox	6.40	0.26	5.90	0.26	300	3
4ljn	7.74	0.29	3.00	0.23	600	6
4qf2	3.21	0.31	3.21	0.31	500	5

*number of models produced with Modeller during PepGrow protocol for the system

Table S6 The structural accuracy of PepGrow represented as the best 1% averages for the full ligand and the first 5 amino acid residues of the ligands for holo systems.

	$RMSD_{best}$		$RMSD_{best,5}$			
PDB ID	**Mean (Å)**	**SD (Å)**	**Mean (Å)**	**SD (Å)**	**# model***	**Docking ranks**
2co0	3.58	0.17	3.58	0.17	500	5
2fuu	6.90	1.45	4.53	0.30	700	7
2ke1	5.35	0.79	3.93	0.20	500	5
2mnz	5.95	0.52	5.13	0.20	500	5
2pvc	4.58	0.90	3.50	0.36	600	6
3o37	6.43	1.58	5.06	0.77	400	4
3sou	4.78	1.03	3.84	0.43	600	6
3qlc	3.54	0.26	3.11	0.29	600	6
4lk9	5.01	0.13	4.07	0.61	300	3
4q6f	3.15	0.21	3.15	0.21	500	5

*number of models produced with Modeller during PepGrow protocol for the system

Table S7a Statistics for docking results with apo proteins

	$RMSD_{top}$		$RMSD_{best}$		$RMSD_{top,5}$[a]		$RMSD_{best,5}$[b]	
**Methods**	**Mean (Å)**	**Std.dev (Å)**	**Mean (Å)**	**Std.dev (Å)**	**Mean (Å)**	**Std.dev (Å)**	**Mean (Å)**	**Std.dev (Å)**
AutoDock 4.2.6	10.88	2.99	8.51	2.53	8.89	1.71	6.60	1.21
CABS-DOCK	15.66	7.96	9.82	3.93	13.73	6.86	8.99	4.28
ClusPro 2.0	15.92	7.40	10.49	4.25	15.02	6.53	8.63	4.71
Gramm-X	14.27	5.68	9.48	3.17	15.24	7.74	7.23	1.67
HADDOCK 2.2	14.76	6.08	10.76	3.31	12.66	4.90	8.33	3.00
HDOCK	18.34	6.48	10.49	4.63	17.20	7.47	7.31	5.86
HPEPDOCK	12.70	6.37	8.41	3.38	10.20	6.31	6.11	2.54
PatchDock	12.55	2.93	9.04	2.71	12.16	4.61	8.34	3.92
PEP-FOLD3	11.79	4.97	9.35	3.86	9.43	3.24	7.32	2.15
PepGrow	13.81	5.97	5.36	1.47	8.49	2.38	4.09	1.18
PiperFlexDock	15.81	9.36	9.66	5.39	13.53	7.83	8.04	4.14

Note: For systems with measured peptide ligands length of five residues, RMSD values calculated for the full length and for the first five were considered to be the same in the calculation of the mean RMSD values

[a] $RMSD_{top,5}$ is the top ranked structure based on the respective interaction energy calculated only for the first 5 amino acids.

[b] $RMSD_{best,5}$ is the best ranked structure based on RMSD calculated only for the first 5 amino acids.

Table S7b Statistics for docking results with holo proteins (For PepGrow, results from E_{inter} ranking were included)

	$RMSD_{top}$		$RMSD_{best}$		$RMSD_{top,5}$[a]		$RMSD_{best,5}$[b]	
**Methods**	**Mean (Å)**	**Std.dev (Å)**	**Mean (Å)**	**Std.dev (Å)**	**Mean (Å)**	**Std.dev (Å)**	**Mean (Å)**	**Std.dev (Å)**
AutoDock 4.2.6	10.99	3.31	7.62	2.39	10.14	3.28	6.77	1.44
CABS-DOCK	13.60	5.97	8.90	3.27	12.71	5.14	6.16	1.81
ClusPro 2.0	17.29	8.41	11.27	5.12	15.29	7.11	9.23	5.61
Gramm-X	15.36	6.34	10.08	4.56	14.95	5.91	8.06	3.81
HADDOCK 2.2	9.39	4.11	8.10	3.11	8.98	5.15	6.60	3.03
HDOCK	11.86	7.64	8.87	4.77	11.51	9.59	6.26	4.72
HPEPDOCK	11.42	5.82	7.61	4.04	7.56	3.16	5.29	2.56
PatchDock	13.42	3.89	10.25	3.75	11.73	4.76	8.63	3.66
PEP-FOLD3	12.94	7.50	7.87	3.12	10.74	5.89	5.86	2.03
PepGrow	11.90	5.08	5.23	1.43	6.48	2.33	3.54	0.69
PiperFlexDock	13.66	9.97	9.01	5.27	11.46	12.05	8.49	11.63

Note: For systems with measured peptide ligands length of five residues, RMSD values calculated for the full length and for the first five were considered to be the same in the calculation of the mean RMSD values

[a] $RMSD_{top,5}$ is the top ranked structure based on the respective interaction energy calculated only for the first 5 amino acids.

[b] $RMSD_{best,5}$ is the best ranked structure based on RMSD calculated only for the first 5 amino acids.

Table S8 Acceptable RMSD thresholds used by benchmark docking methods.

Method	RMSD Threshold (Å)
PIPER-FlexPepDock	L-RMSD[a] ≤ **3.0**
CABS-DOCK	RMSD < **3.0**
ClusPro 2.0	L-RMSD[a] < **10.0**
HADDOCK	L-RMSD[a] ≤ 2.**0**
GRAMM-X	N/A
HDOCK	N/A
HPEPDOCK	I-RMSD[b] **≤ 2.0**
PatchDock	N/A
PEP-FOLD3	N/A
**Average**	**4.0**
**SD**	**3.0**

[a] L-RMSD: "the backbone root-mean-square displacement of the ligands in the predicted versus the target structures computed after the receptors of these structures have been superimposed"
[b] I-RMSD: the same as L-RMSD, but only for the interacting part of the ligand

Table S9 Average RMSD of top 1% results obtained with PepGrow for apo and holo proteins when ranked according to E_{inter} and its components, respectively.

PDB ID	E_{inter}(total)				E_{inter}(Lennard-Jones)				E_{inter}(Coulomb)			
	**APO**											
	$RMSD_{top}$ (Å)	SD (Å)	$RMSD_{top,5}$[a] (Å)	SD (Å)	$RMSD_{top}$ (Å)	SD (Å)	$RMSD_{top,5}$[a] (Å)	SD (Å)	$RMSD_{top}$ (Å)	SD (Å)	$RMSD_{top,5}$[a] (Å)	SD (Å)
1xwh	11.36	4.80	7.59	2.64	16.82	7.87	8.87	4.70	12.55	6.65	6.65	1.11
2fui	20.10	4.23	9.42	1.09	19.66	4.10	7.76	2.73	16.74	4.58	7.51	1.97
2gnq	8.04	1.45	8.04	1.45	7.78	1.56	7.78	1.56	6.74	1.59	6.74	1.59
2mny	19.44	2.51	7.31	1.61	20.12	3.36	8.66	0.19	16.63	1.72	6.80	1.08
2pv0	9.38	3.05	7.33	1.92	9.06	3.05	7.97	1.69	13.62	1.31	10.26	1.76
3o33	23.79	1.66	12.57	1.75	24.88	1.26	13.49	0.34	21.55	5.75	11.29	2.62
3qln	13.70	0.87	9.49	0.89	13.13	0.61	9.57	0.15	12.81	1.67	8.42	0.92
3sox	12.59	1.95	8.34	1.50	12.14	1.37	9.43	0.81	9.05	1.43	8.34	0.47
4ljn	11.52	1.42	3.54	0.90	12.21	4.95	5.26	2.25	13.36	3.04	5.51	1.90
4qf2	8.80	2.47	8.80	2.47	10.65	3.13	10.65	3.13	8.81	2.45	8.81	2.45
	**HOLO**											
2co0	7.13	1.40	7.13	1.40	7.57	1.00	7.57	1.00	8.65	2.47	8.65	8.65
2fuu	17.23	6.30	7.37	1.34	19.36	3.69	10.17	1.74	20.57	5.32	8.43	20.57
2ke1	13.16	2.41	7.68	2.64	15.58	0.78	8.74	0.99	13.36	1.40	7.82	13.36
2mnz	11.59	4.68	6.93	1.01	15.07	5.47	8.24	2.13	17.23	6.60	8.79	17.23
2pvc	7.61	1.26	4.55	0.76	8.82	2.41	6.28	1.50	7.84	1.89	4.63	7.84
3o37	24.59	1.26	11.46	0.70	23.42	1.42	10.64	2.03	20.21	3.84	8.77	20.21
3sou	11.15	3.46	9.05	3.16	12.12	2.57	2.85	2.67	9.92	2.09	7.44	9.92
3qlc	9.06	3.85	5.95	2.74	10.81	1.67	8.13	2.07	9.79	3.01	6.83	9.79
4lk9	10.04	0.95	6.84	1.40	9.20	1.18	6.38	1.47	10.05	1.90	8.30	10.05
4q6f	6.61	4.11	6.61	4.11	6.47	4.15	6.47	4.15	5.79	1.71	5.79	5.79

[a] $RMSD_{top,5}$ is the top ranked structure based on the respective interaction energy calculated only for the first 5 amino acids.

Table S10 RMSD values of target-target superimposition of apo to holo targets of the same systems.

**Holo PDB ID**	**2co0**	**2fuu**	**2ke1**	**2mnz**	**2pvc**	**3o37**	**3qlc**	**3sou**	**4lk9**	**4q6f**
**Apo PDB ID**	**2gnq**	**2fui**	**2xwh**	**2mny**	**2pv0**	**3o33**	**3qln**	**3sox**	**4ljn**	**4qf2**
RMSD of superimposition (Å)	0.18	0.54	0.70	1.65	0.55	0.31	0.32	0.41	0.43	0.23

Table S11 The effect of random seed number variation on the results of the homology modelling by MODELLER program package, based on its built in scoring function.

	Default seed number		Seed number = -200		Seed number = -2000		Seed number = -20000	
**Model No.**	**molpdf**	**DOPE score**	**molpdf**	**DOPE score**	**molpdf**	**DOPE score**	**molpdf**	**DOPE score**
1	318.60	-4933.76	338.43	-5009.15	339.47	-4911.88	354.55	-4991.48
2	415.39	-4710.17	347.51	-4923.44	318.19	-4890.90	341.69	-5035.76
3	347.36	-5033.81	356.04	-4993.34	352.56	-4893.37	332.33	-4898.70
4	358.58	-4945.49	352.01	-4924.98	339.19	-4997.85	333.47	-4868.92
5	330.26	-4927.45	339.98	-5005.39	333.64	-4873.16	365.22	-4950.63
6	383.37	-4858.96	394.88	-4809.63	323.94	-5004.62	348.19	-4860.29
7	344.71	-4940.15	343.92	-4868.42	348.07	-4877.93	335.21	-4955.34
8	358.14	-4927.16	326.20	-4919.68	332.21	-4915.46	365.75	-4988.92
9	370.40	-4857.71	344.34	-4912.85	368.51	-4878.62	338.88	-4989.56
10	332.76	-4996.95	332.53	-4917.87	368.28	-4962.81	354.12	-4949.08
11	358.92	-4885.64	324.95	-4970.97	351.62	-4992.89	356.44	-4889.34
12	358.40	-4949.82	356.86	-4930.74	333.81	-4936.94	343.28	-4942.15
13	332.87	-4945.93	325.19	-5039.16	343.46	-4954.72	348.26	-4988.04
14	449.52	-4561.36	352.70	-4909.69	375.33	-4905.00	338.87	-4929.67
15	340.83	-4900.82	350.65	-4907.11	343.06	-4973.36	350.09	-4926.46
16	368.71	-4957.64	352.83	-4923.73	351.95	-4968.50	346.47	-4971.17
17	333.62	-4955.68	337.39	-4912.79	378.40	-4943.83	366.96	-4972.85
18	353.19	-4988.81	334.97	-4879.81	382.83	-4905.95	350.86	-4812.19
19	383.81	-4758.42	385.61	-4975.10	343.51	-5031.80	352.36	-4899.20
20	338.68	-4977.28	344.33	-4941.53	336.64	-4867.25	336.98	-4971.76

Table S12 Results of ranking according to the E_{inter} calculated for the full length peptides (E_{inter} ranking) and for the first five residues of peptides ($E_{inter,5}$ ranking) resulted from PepGrow with apo proteins and the representative structures of the top 1%.

PDB ID	E_{inter} ranking		Representatives		$E_{inter,5}$ ranking		Representatives	
	$RMSD_{top}$	$RMSD_{top,5}$[a]	$RMSD_{top}$	$RMSD_{top,5}$[a]	$RMSD_{top}$	$RMSD_{top,5}$[a]	$RMSD_{top}$	$RMSD_{top,5}$[a]
1xwh	11.48	6.66	11.48	6.66	13.20	6.64	8.56	5.83
2fui	20.66	9.05	20.66	9.05	13.66	10.38	17.75	8.74
2gnq	8.10	8.10	8.21	8.21	8.10	8.10	8.21	8.21
2mny	20.70	8.73	18.33	6.36	16.54	5.53	18.33	6.36
2pv0	10.54	7.50	9.05	5.79	10.54	7.50	9.51	8.46
3o33	23.47	13.53	23.90	12.30	24.56	13.19	27.18	13.64
3qln	13.28	9.51	13.28	9.51	13.28	9.51	13.28	9.51
3sox	10.57	8.51	10.57	8.51	14.47	6.77	10.32	8.78
4ljn	10.35	4.99	11.90	4.15	10.27	3.59	15.02	5.42
4qf2	8.25	8.25	9.99	9.99	8.25	8.25	9.99	9.99

[a] $RMSD_{top,5}$ is the top ranked structure based on the respective interaction energy calculated only for the first 5 amino acids.

Table S13 Scoring functions of the benchmark methods tested in the present study.

**Method and formula of scoring function**	**Explanation of formula**
AutoDock 4.2.6	
$\Delta G = (V_{bound}^{L-L} - V_{unbound}^{L-L}) + (V_{bound}^{P-P} - V_{unbound}^{P-P}) + (V_{bound}^{P-L} - V_{unbound}^{P-L} + \Delta S_{conf})$<br>$V = W_{vdw} \sum_{ij} \left(\frac{A_{ij}}{r_{ij}^{12}} - \frac{B_{ij}}{r_{ij}^{6}} \right) + W_{hbon} \sum_{ij} E(t) (\frac{C_{ij}}{r_{ij}^{12}} - \frac{D_{ij}}{r_{ij}^{12}})$<br>$+ W_{elec} \sum_{ij} \frac{q_i q_j}{\varepsilon(r_{ij}) r_{ij}} + W_{sol} \sum_{ij} (S_i S_j + S_j S_i) e^{(-r_{ij}^2/2\sigma^2)}$	V; pairwise atomic term calculated between unbound and bound states of ligand (L) and target (P) molecules, ΔS_{conf} ; conformational entropy loss upon binding<br>W_{vdw}; weighting constant for Lennard-Jones 6/12 potential, A and B; force field parameters, i; ligand atoms, j; protein atoms, r_{ij}; interatomic distance<br>W_{hbond} ; weighting constant for hydrogen bonding term based on a 10/12 potential with E(t), the directional angle-based weight depending on angle t accounting for ideal H-bonding geometry, C and D; force field parameters<br>W_{elec} ; weighting constant for Coulombic electrostatic potential, q; atomic partial charges, $\varepsilon(r_{ij})$; a distance-dependent dielectric function<br>W_{sol} ; weighting constant for de-solvation term, V; fragmental volumes of atoms j surrounding a given atom i, S; a solvation parameter, σ; a scaling constant
CABS-dock	
$E = E_g + 0.375 \times E_s + E_H + E_R + 2E_P$<br>**Term 1:** $E_G = \sum(B_B + B_S + B_H + B_E + B_{HH} + B_{EE} + B_C)$<br>**Term 2:** $E_s = \Sigma(0.5 \times E_{13} + E_{14} + E_{15})$<br>$E_{13} = E_{13}(\lvert r_3 - r_1 \rvert, A_3, A_1)$<br>$E_{14} = E_{13}(\lvert r_4 - r_1 \rvert, A_3, A_2)$<br>$E_{15} = E_{13}(\lvert r_5 - r_1 \rvert, A_4, A_2)$<br>**Term 3:** $E_H = \Sigma\Sigma(g_{i,j} \times E_h)$<br>$E_h = \delta_h \times \varepsilon_h \times (1.0 + (4.25/max\{4.25, min\{6.01, r_{pp}\}\}))^4 - 0.25 + ((4.25/max\{4.25, min\{6.01, r_{qq}\}\})^4 - 0.25) + \delta_\gamma \times \varepsilon_\gamma \times (2.0 - max\{(b_i \cdot (r_i - r_j)/6.1)^2, 0.125\} - max\{(b_j \cdot (r_i - r_j)/6.1)^2, 0.125\}$<br>**Term 4:** $E_R = \Sigma(E_{C_\alpha - C_\alpha} + E_{C_\alpha - pb} + E_{C_\alpha - SG})$<br>$E_{C_\alpha - C_\alpha} = \varepsilon_r \times \left(\left(\frac{3.05}{d_{C_\alpha - C_\alpha}} \right)^2 - 0.5 \right)$<br>$E_{C_\alpha - pb} = \varepsilon_r \times ((4.23/max\{4.23, d_{C_\alpha - pb}\})^2 - 0.75)$<br>$E_{C_\alpha - SG} = \varepsilon_r \times ((3.66/max\{4.48, d_{C_\alpha - SG})^2 - 2/3)$<br>**Term 5:** $E_P = \Sigma(\varepsilon(A_{i'} A_{j'} \Theta_{i'} \Theta_{j'} \Phi_{i,j}) \times (D_{max}(A_{i'} A_{j'} \Theta_{i'} \Theta_{j'} \Phi_{i,j})/d_{i,j})^2$	**Term 1:** E_g ; short range sequence-independent interaction term, B_B; bias towards protein-like chain stiffness, B_S ; local protein-like chain stiffness, B_H ; bias towards helical secondary structure, B_E ; bias towards β-strands, B_{HH} ; bias towards the same helical or expanded secondary structure, B_{EE} ;bias towards the same β-strands, B_C ; bias against crumpled structures<br>**Term 2:** E_S ; short range sequence-dependent interaction term, A_i ; amino acid at i-th position, r_i; distance between residues at i-th position<br>**Term 3:** E_H ; hydrogen bonds term, g_{ij}; factor for hydrogen bonds in regular secondary structures, r_{pp} and r_{qq} ; proper distance between the center of peptide bonds, ε_h and ε_γ; optimized scaling factors, δ_h and δ_γ, scaling factors which determines if geometrical conditions are satisfied or not<br>**Term 4:** E_R ; repulsive interactions term, $E_{C_\alpha - C_\alpha}$; repulsive interactions between Cα atoms along the sequence, d; distance between the respective atoms, $E_{C_\alpha - pb}$; those between Cα atoms and centers of peptide bonds, $E_{C_\alpha - SG}$; those between Cα atoms and their side-chains, ε_r; scaling factor for repulsive interactions which is 5<br>**Term 5:** E_P ; long range pairwise interactions term, A_i and A_j; identity of i and j amino acids, Θ_i and Θ_j ; values defining conformation of the i and j amino acids, Φ_i and Φ_j ; value defining orientations of side chains of the i and j amino acids
ClusPro	
$E = E_{shape} + w_2 E_{elec} + w_3 E_{DARS}$<br>**Term 1:** $R_p(l,m,n) = -c_{l,m,n} + w_1 r_{l,m,n}$<br>$L_p(l,m,n) = \begin{cases} 1 \ if \ (l,m,n) \ni (a_j \in J) \\ 0 \quad otherwise \end{cases}$<br>**Term 2:** $E_{elec} = \sum_{i=1}^{N_R} \sum_{j=1}^{N_L} \frac{q_i q_j}{\left(r_{ij}^2 + D^2 \exp\left(\frac{-r_{ij}^2}{4D^2} \right) \right)^{\frac{1}{2}}}$<br>**Term 3:** $E_{DARS} = \sum_{i=1}^{N_R} \sum_{j=1}^{N_L} \varepsilon_{ij}$	w_1, w_2 and w_3 ; scaling factors<br>**Term 1:** E_{shape}, shape complementary term defined by repulsive (R_p) and attractive (L_p) interactions, $c_{l,m,n}$; the number of atoms at the distance of d < r < D from the grid point (l,m,n) in which D is the cut off value of attractive interactions (6Å) and d is the cut off value of repulsive interactions (d = r_{vdw} + 2 Å), $r_{l,m,n}$; the number of atoms at the distance of r < d from the grid point (l,m,n)<br>**Term 2:** E_{elec}; electrostatic energy term, N_R and N_L; the number of atoms in the ligand and the receptor, r_{ij} ; distance between i and j atoms, q_i and q_j; charges of i and j atoms

$\varepsilon_{IJ} = \sum_{p=1}^{K} \lambda_p u_{pI} u_{pJ}$	**Term 3:** E_{DARS}; decoys as the reference state pairwise term, ε_{ij}; the interacting energy between atoms of i and j types, K; number of different atom types, λ_p; p-th eigenvalue of the interacting matrix, u_{pI} and u_{pJ} ; I-th and J-th components of the p-th eigenvector
Gramm-X	
$V_{ij}(r) = \frac{1}{\alpha\sigma_{ij}^{6} + r^6}\left(\frac{4\varepsilon_{ij}\sigma_{ij}^{12}}{\alpha\sigma_{ij}^{6} + r^6} - 4\varepsilon_{ij}\sigma_{ij}^{6}\right)$	r; distance between i and j atoms, α = 0.4, σ; distance at which V = 0 and is equal to 0.33 nm, ε; dispersion energy and is equal to 0.5
HADDOCK	
HADDOCKscore-it0 = 0.01 E_{vdw} + 1.0 E_{elec} + 1.0 $_{desol}$ + 0.01 E_{air} - 0.01 BSA<br>HADDOCKscore-it1 = 1.0 E_{vdw} + 1.0 E_{elec} + 1.0 E_{desol} + 0.1 E_{air} - 0.01 BSA<br>HADDOCKscore-water = 1.0 E_{vdw} + 0.2 E_{elec} + 1.0 E_{desol} + 0.1 E_{air}<br>**Term 1:** $E_{vdw} = \frac{A_{ij}}{d_{ij}^{12}} - \frac{B_{ij}}{d_{ij}^{6}}$<br>**Term 2:** $E_{elec} = \frac{pq}{K\zeta}\left[\frac{1}{d} + \frac{(\zeta-\epsilon)/(\zeta+\epsilon)}{\sqrt{d^2+4s_p s_q}}\right]$<br>**Term 3:** $E_{desol} = \sum_i \Delta\sigma_i A_i$<br>**Term 4:** $E_{air} = \sum_i d_{iAB}^{eff}$<br>$d_{iAB}^{eff} = \left(\sum_{m_{iA}=1}^{N_{atoms}} \sum_{k=1}^{N_{resB}} \sum_{n_{kB}=1}^{N_{atoms}} \frac{1}{d_{m_{iA}n_{kB}}^{6}}\right)^{-\frac{1}{6}}$	**Term 1:** E_{vdw}; van der Waals intermolecular energy, A_{ij} and B_{ij}; force field parameters for the Lennard-Jones 6/12 potential, d_{ij}; interatomic distance<br>**Term 2:** E_{elec}; electrostatic intermolecular energy, p and q; electrostatic charges on the probe group and pairwise protein atom, K; geometrical factor, d; the distance between the probe group and pairwise protein atom, ζ and ϵ; effective dielectric values for the protein and the solution s_p and s_q; depth of the probe at each x, y, z positions and depth of each protein atom,<br>**Term 3:** E_{desol}; desolvation energy, σ_i; atomic solvation parameter, A_i; Lee and Richards solvent-accessible surface area for the atom<br>**Term 4:** E_{air}; ambiguous interaction restraints energy calculated for each active or passive residues, d_{iAB}^{eff}; effective distance, A and B; interacting molecules, m_{iA}; any *m* atom of an *i* active residue of protein A , n_{kB}; any n atom of *k* both active and passive residues of protein B, N_{atoms}; all atoms of a residue, N_{res}; all active and passive residues of a protein BSA; buried surface area,
HDOCK	
$u_{ij}^{k+1}(r) = u_{ij}^{k}(r) + \Delta u_{ij}^{k}(r)$<br>$\Delta u_{ij}^{k}(r) = \frac{1}{2}k_B T[g_{ij}^{k}(r) - g_{ij}^{obs}(r)]$	k; iterative step, i and j; types of a pair of atoms in the receptor and the ligand, $u_{ij}^{k}(r)$; the pair potential between atom types of i and j at r distance at the k-th iteration step, $u_{ij}^{k+1}(r)$; an improved potential from $u_{ij}^{k}(r)$, k_B; the Boltzmann constant, T; the system temperature, $g_{ij}^{obs}(r)$; the pair distribution function of i and j atom pair observed in the experimentally determined protein-protein complexes, $g_{ij}^{k}(r)$; the pair distribution function of i and j atom pair calculated from possible interaction modes
HPEPDOCK	
$energy\ score = \sum_{P-L atom pair} u_{ij}(r)$	P-L atom pair; all pairs of the protein and the ligand atoms, $u_{ij}(r)$; the pair potential between atom types of i and j at r distance calculated from 786 protein-ligand complexes
PatchDock	
During geometric scoring, the receptor is represented as distance transform grids and is divided into 5 shells according to the distance from the surface. The geometry score is a weighted function of ligand surface points in each shell and ligand poses with a high number of points in the exterior shell (-1.0, 1.0) and as few as possible number points in the interior shells are scored higher.	
PEP-FOLD3	

$E = E_{local} + E_{nonbonded} + E_{H-bond}$<br>**Term 1:** $E_{\text{local}} = w_b \sum_{\text{bonds}} K_b(r - r_{eq})^2 + w_a \sum_{\text{angles}} K_\alpha(\alpha + \alpha_{eq})^2 + w_\Omega \sum_{\text{imp-torsions}} K_\Omega(\Omega + \Omega_{eq})^2 + w_{\Phi,\Psi}(\sum_\Phi E_\Phi + \sum_\Psi E_\Psi)$<br>$E_\Phi = k_{\Phi\Psi}(\Phi - \Phi_0)^2$<br>$E_\Psi = k_{\Phi\Psi}(\Psi - \Psi_0)^2$<br><br>**Term 2:** $E_{nonbonded} = w_{1,4}\sum_{1,4} E_{VdW} + w_{C\alpha,C\alpha}\sum_{C\alpha,C\alpha} E_{VdW} + w_{1>4}\sum_{M',M'} E_{VdW} + w_{1>4}\sum_{M',C\alpha} E_{VdW} + w_{1>4}\sum_{M',Sc} E_{VdW} + w_{Sc,Sc}\sum_{Sc,Sc} E_{VdW}$<br>$E_{VdW} = \varepsilon_{ij}\left(\left(\frac{r_{ij}^o}{r_{ij}}\right)^{12} - 2\left(\frac{r_{ij}^o}{r_{ij}}\right)^6\right)H(\varepsilon_{ij}) - \varepsilon_{ij}\left(\frac{r_{ij}^o}{r_{ij}}\right)^6 H(-\varepsilon_{ij})$<br>$r_{ij}^0 = (r_i^0 + r_j^0)/2$<br><br>**Term 3:** $E_{HB1} = w_{hb1-4}\sum_{ij,j=i+4}\varepsilon_{hb1-4}\mu(r_{ij})\upsilon(\alpha_{ij}) + w_{hb1>4}\sum_{ij,j>i+4}\varepsilon_{hb1>4}\mu(r_{ij})\upsilon(\alpha_{ij})$<br>$\mu(r_{ij}) = 5\left(\frac{\sigma}{r_{ij}}\right)^{12} - 6\left(\frac{\sigma}{r_{ij}}\right)^{10}$<br>$\nu(\alpha_{ij}) = \begin{cases} cos^2\alpha_{ij}, \alpha_{ij} > 90^0 \\ 0, \quad otherwise \end{cases}$	**Term 1:** E_{local}; local energy term, w_b,w_a,w_Ω and $w_{\phi,\psi}$; force constants related to changes in bond lengths, angles, improper torsions and torsional angles, K_b,K_a,and K_Ω; force constants associated with the main chain atoms, r; bond length, α; bond angle, *Ω*; improper torsion angle, r_{eq}, α_{eq} and Ω_{eq}; the respective equilibrium values, E_Φ and E_Ψ; potentials related to dihedral angles,Φ and Ψ; dihedral angles, $k_{\Phi\Psi}$; force constant related to dihedral angles<br>**Term 2:** $E_{nonbonded}$; nonbonded energy term, $w_{1,4}$, $w_{C\alpha,C\alpha}$, $w_{1>4}$ and $w_{M',M'}$; force constants, 1,4; 1-4 interactions along each torsional degree, 1>4; long range interactions, Cα; alpha carbon atoms, M`; main chain atoms, Sc; side chain atoms, r_{ij}; distance between i and j atoms, $r_i^0$and r_j^0; Van der Waals radius of i and j atoms, ε_{ij} ; dispersion energy between i and j atoms, H(x); heavy side function<br>**Term 3:** E_{HB1}; two-body hydrogen bond energy term, w_{hb1-4} and $w_{hb1>4}$; force constants of short-range and long-range H-bonds respectively, the sum includes all residues i and j where j is separated from i by 4 residues (j = i + 4), ε_{hb1-4} and $\varepsilon_{hb1>4}$; short and long-range H-bond energies, r_{ij}; distance between the carbonyl oxygen and amide hydrogen of i and j residues, $\boldsymbol{\alpha}_{ij}$; angle between NHO atoms, $\boldsymbol{\sigma}$; the equilibrium distance between O and H atoms
PiperFlexDock	
Energy for body-rigid docking with PIPER:<br>$E = E_{shape} + w_2E_{elec} + w_3E_{DARS}$<br>$R_p(l,m,n) = -c_{l,m,n} + w_1r_{l,m,n}$<br>$L_p(l,m,n) = 1 \; if \; (l,m,n) \ni (a_j \in J)$<br>= 0 otherwise<br>$E_{elec} = \sum_{i=1}^{N_R}\sum_{j=1}^{N_L}\frac{q_iq_j}{\left(r_{ij}^2 + D^2\exp\left(\frac{-r_{ij}^2}{4D^2}\right)\right)^{\frac{1}{2}}}$<br>$E_{pair} = \sum_{i=1}^{N_R}\sum_{j=1}^{N_L}\varepsilon_{ij}$<br>$\varepsilon_{IJ} = \sum_{p=1}^{K}\lambda_p u_{pI}u_{pJ}$<br>Refinement scoring function:<br>$E = E_{rama} + E_{LJ} + E_{hb} + E_{sol} + E_{pair} + E_{dun} + E_{ref}$<br>**Term 1:** $E_{rama} = \sum_i -\ln[P(\Phi_i, \Psi_i \vee aa_i, ss_i)]$<br>**Term 2:**<br>$E_{LJ} = \begin{cases} \left[\left(\frac{r_{ij}}{d_{ij}}\right)^2 - 2\left(\frac{r_{ij}}{d_{ij}}\right)^6\right]e_{ij}, \text{if } \frac{d_{ij}}{r_{ij}} > 0.6 \\ \left[-8759.2\left(\frac{d_{ij}}{r_{ij}}\right) + 5672.0\right]e_{ij}, \text{else} \end{cases}$<br><br>**Term 3:** $E_{hb} = \sum_i\sum_i(-\ln[P(d_{ij} \mid h_iss_{ij})] - \ln[P(\cos\theta_{ij} \mid d_{ij}h_jss_{ij})] - \ln[P(\cos\psi_{ij} \mid d_{ij}h_jss_{ij})])$<br>**Term 4:** $E_{sol} = \sum_i\left[\Delta G_i^{ref} - \sum_i\left(\frac{2\Delta G_i^{ref}}{4\pi^{3/2}\lambda_i r_{ij}^2}e^{-d_{ij}^2}V_j + \frac{2\Delta G_i^{free}}{4\pi^{3/2}\lambda_i r_{ij}^2}e^{-d_{ij}^2}V_j\right)\right]$	Energy for body-rigid docking with PIPERq:<br>w_1, w_2 and w_3 ; scaling factors<br>**Term 1:** E_{shape}, shape complementary term, $c_{l,m,n}$; the number of atoms at the distance of d < r < D from the grid point (l,m,n) in which D is the cut off value of attractive interactions (6Å) and d is the cut off value of repulsive interactions (d = r_{vdw} + 2 Å), $r_{l,m,n}$; the number of atoms at the distance of r < d from the grid point (l,m,n)<br>**Term 2:** E_{elec}; electrostatic energy term, N_R and N_L; the number of atoms in the ligand and the receptor, r_{ij} ; distance between i and j atoms, q_i and q_j; charges of i and j atoms, D; an atom type independent approximation of the generalized Born radius<br>**Term 3:** E_{pair}; a statistical pairwise potential , ε_{ij}; a pairwise interaction potentail between atoms of i and j, K; number of different atom types, λ_p; p-th eigenvalue of the interacting matrix, u_{pI} and u_{pJ} ; I-th and J-th components of the p-th eigenvector<br>Refinement scoring function:<br>**Term 1:** E_{rama}; Ramachandran torsional term, i; residues, Φ_i and Ψ_i; backbone torsional angle, aa_i; amino acid type, ss_i; secondary structure type<br>**Term 2:** E_{LJ}; Lennard-Jones interaction term, i and j, residues, r_{ij}; sum van der Waals radii, d_{ij}; interatomic distance, e_{ij}; geometric mean of atom well depths taken from CHARMm19<br>**Term 3:** E_{hb}; hydrogen bonds term, i and j; donor and acceptor residues, d_{ij}; interatomic distance between donor and proton, ss_{ij}; secondary structure type, θ_{ij}; bond angle between protein-acceptor-acceptor, Ψ_{ij}; bond angle between donor-proton-acceptor<br>**Term 4:** E_{sol}; solvation term, i and j, atom indices, ΔG^{ref} and ΔG^{free}; energy of fully solvated atoms, λ_i; correlation length, r_{ij}; sum of van der Waals radii, d_{ij}; interatomic distance, V_j;

**Term 5:** $E_{pair} = \sum_i \sum_{j>i} -\ln\left[\frac{P(aa_i, aa_i \mid d_{ij})}{P(aa_i \mid d_{ij}) P(aa_i \mid d_{ij})}\right]$<br>**Term 6:** $E_{dun} = \sum_i -\ln\left[\frac{P(rot_i \mid \Phi_i, \Psi_i) P(aa_i \mid \Phi_i, \Psi_i)}{P(aa_i)}\right]$<br>**Term 7:** $E_{ref} = \sum_{aa} n_{aa}$	atom volume<br>**Term 5:** E_{pair}; residue pair interactions term, aa_i; amino acid type, d_{ij}; distance between residues<br>**Term 6:** E_{dun}; rotamer self-energy term, rot_i; Dunbrack backbone-dependent rotamer, aa_i; amino acid type, Φ_i and Ψ_i; backbone torsional angles<br>**Term 7:** E_{ref}; unfolded state reference energy term, n_{aa}; number of residues, aa, amino acid type

Figures

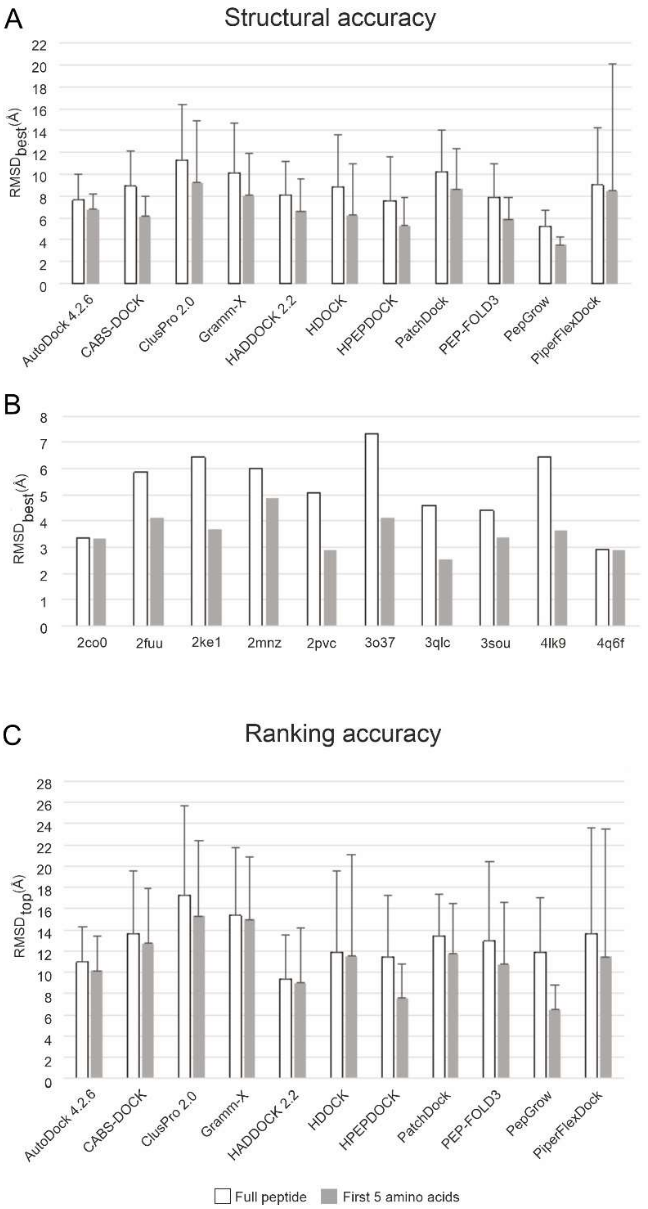

Figure S1 The statistics of docking results obtained for all test systems of Table S1 using all holo target structures. A) Columns represent the mean of $RMSD_{best}$ values (of all test systems) calculated for ligand binding modes supplied by PepGrow and the 10 benchmark methods, respectively. Error bars represent standard deviations (see also Table S7b). B) Structural performance of PepGrow on the individual test systems (see also Table S6). C) Columns represent the mean of $RMSD_{top}$ values (of all test systems) calculated for ligand binding modes supplied by PepGrow and the 10 benchmark methods, respectively. Error bars represent standard deviations (Table S7b).

Supplementary Methods

Application of benchmark methods

General settings. All 10 external benchmark methods were freely accessible as a web server or standalone program (AutoDock 4.2.6). File type of the inputs used in our study and outputs for individual docking servers are described below. All docking methods were run with their default parameters unless noted below. To ensure an unbiased comparison of the docking methods, target and ligand input files, and binding pocket information were provided in a standardized way for all methods. The centre of the binding pocket of the target in AutoDock 4.2.6 was determined by the geometrical centre (or average coordinates) of the experimental ligand structure. In other methods, the binding site information was provided as potential binding residues in the peptide and the target. A central ligand residue containing the central atom of the experimental ligand conformation was selected. On the target side, a residue that has the closest heavy atom to the central ligand residue was selected as a potential binding site residue.

Preparation of targets and ligands. Both apo and holo forms of the proteins were used as targets in respective docking trials of all benchmark methods. The apo target protein structures were chosen and adequately prepared for the requirements of the servers. Atomic coordinates of the holo and apo target proteins (Table 1) were downloaded from the PDB. A "clean" target structure was produced by removing all non-protein parts, including the peptides ligands and all solvent molecules, except important metal ions in the binding site. Swiss-Model [29] was employed to detect and reconstruct missing residues and/or atoms in the target structure. The error-free, full sequence of the protein was provided obtained from UniProt [30]. Pymol was used to add capping groups and to align the apo targets to their holo counterparts. No further minimization and structural optimization were performed on the final protein structure to retain the original coordinates of all heavy atoms of the crystallographic PDB structure. Low energy ligand conformations were built and optimized with the TINKER software package [31]. The peptide structures were built from their experimentally determined sequence without specific protonation states or angles using the 'protein' command with Amber99 force field. In cases of peptides with missing terminal amino acids, capping groups were added. The resulting structures were further minimized with AMBER ff99 force field [32] and RMS Gradient per Atom Criterion of 0.001 using the 'newton' command. Finally, the xyzpdb command was employed to convert the minimized structure file in XYZ format into PDB format.

Specific settings. **AutoDock 4.2.6** AutoDockTools4 [19] was used to prepare input PDBQT files where adding polar hydrogen atoms and Gasteiger-Marsilli partial charges [33]. The target was considered a rigid body while for peptides, torsional degrees of freedom were set to enable peptide backbone rotations. Prior to docking, the grid affinity calculations were performed by AutoGrid4 where the coordinates of the grid box centre were set as the average coordinates of the experimental ligand structure. The grid box size, defined as the number of grid points (N_{pts}), was calculated using **Eq. 2**.

$$N_{pts} = (L_{max} + T) / G \qquad \textbf{Eq. 2}$$

where L_{max} is the maximal length of the ligand measured between the farthest two heavy atoms in the ligand, T is a distance tolerance (10 Å) between the edge of the docking box and the ligand and G is the grid spacing which was set as 0.375 Å. Docking was carried out using the Lamarckian Genetic Algorithm and the number of docking runs was set to 10 with the maximum number of evaluations of 25 million. As AutoDock 4.2.6 does not handle ligands with more than 32 torsion angles, for larger peptides a recompiled version allowing up to 64 torsion angles was used. Top 10 ligand positions were acquired, structurally clustered (tolerance of 2 Å), and ranked according to their AutoDock 4.2.6 binding free energy values as it was described previously [34]. Briefly, the ligand structure with the lowest calculated free energy of binding was selected, and the neighbouring docked ligand structures within 2 Å were collected in the rank, then a new rank is opened starting with an unused structure of the lowest calculated free energy of binding from the remaining structures, etc. until all ligand structures were collected into ranks.

CABS-DOCK applies the coarse-grained CABS protein representation [35] in which each amino acid is represented by up to four interaction centres. First, randomly generated peptide conformations are placed on the surface of the receptor's geometrical centre followed by Replica Exchange Monte Carlo dynamics

with 10 replicas uniformly spread on the temperature scale. During the simulation, distance restraints were applied on C-alpha atom pairs within 5-15 Å on the receptor, the minimum sequence gap between restrained residues is set to 5. From the resulting trajectories, 100 lowest energy structures are selected and clustered using the k-medoids procedure with different initial medoids 100 times. Ten consensus medoids are selected and their all-atom representations are modelled followed by further refinement using Modeller with DOPE statistical potential [36]. As input, the target structure in PDB format and the amino acid sequence of the peptide in one letter format was provided. In the optional settings, the potential binding residues of the ligand and the target were specified and the remaining options were left empty. Thus no target residues were selected for marking flexible regions and unlikely to bind regions of the ligand. The peptide secondary structure was not defined thus by default PSIPRED method is automatically used. The default contact cut-off distance between the heavy atoms of the residues is 5 Å and the weight factor for distance above the cut-off is 1. By default, 50 Monte Carlo simulation cycles were set and 1000 binding modes were clustered into 10 clusters. As a result, a compressed file containing the structure of all members of the 10 clusters and detailed statistics of the top 10 binding modes as well as their contact information were provided on the results page.

ClusPro 2.0 performs rigid body docking by PIPER, a FFT correlation approach-based docking program with [37]. The 1000 lowest energy docked structures are clustered based on their interface RMSD values (tolerance of 9 Å) followed by energy minimization of the selected structures with fixed backbone using only the van der Waals term of Charmm potential. Finally, the centres of the 10 most populated clusters are retained. Since non-standard amino acids and heteroatoms are not accepted, the ligand and the target structures were provided without zinc ions and capping groups in PDB format as input. All the advanced options were not specified. By default, the lowest energy 1000 binding modes were structurally clustered and the cluster representatives were ranked according to four different scoring schemes which represent differences in the biophysical forces that dominate interactions between protein and peptide. In this study, we chose to use the "balanced" scoring scheme.

GRAMM-X performs FFT-based rigid-body docking with a smoothed Lennard-Jones potential followed by the local minimization and re-scoring with knowledge-based potential terms. As input, the ligand and the target structures without zinc ions and capping groups in PDB format were submitted and the potential binding residues were specified in the optional parameters. By default, 30000 docking run was performed followed by structural clustering with tolerance of 10 Å and the top 10 binding modes were set to be saved in the final output file.

HADDOCK 2.2 is an ambiguous interaction restraints (AIRs)-guided rigid-body docking method. Active residues were determined from the experimental structure as described in Section General settings. For each active residue, an AIR restraint is defined based on an effective distance between any atom of an active residue and any atom of all active and passive residues on the partner molecule (more from Table S). The docking consists of three stages: (1) rigid body energy minimization (it0); (2) semi-flexible refinement (it1); (3) final refinement with explicit solvent. First, random orientations of the two molecules are produced by positioning them at 150 Å from each other and randomly rotating them around their mass centre. It was followed by rigid body energy minimization in which both translational and rotational movements are allowed. The top-ranked 200 conformations according to their HADDOCKscore-it0 are then retained for further refinement. In the second stage, the semi-flexible refinement is performed with three simulated annealing MDs and their default values are (i) 500 MD steps from 2000 to 500 K treating two molecules as rigid bodies to optimize their orientations, (ii) 1000 steps from 1000 to 50 K with flexible the side chains at the interface, (iii) 1000 steps from 1000 to 50 K with fully flexible interface. Time steps are set to 2 fs for all MDs. In the final stage, a refinement with TIP3P water molecules is performed and the default values at each phase are: (i) heating phase, 100 steps at 100, 200, and 300 K with position restraints of 5 kcal mol^{-1} A^{-2} on all atoms except for the side chains at the interface, (ii) 1250 MD steps at 300 K with position restraints of 1 kcal mol^{-1} A^{-2} on only non-interface heavy atoms, (iii) cooling stage, 500 MD steps at 300, 200, and 100 K with the position restraints are limited to backbone atoms outside the interface. The resulting solutions are clustered according to their backbone RMSD values at the

interface (tolerance of 1 Å) and the clusters are ranked according to their HADDOCK scores. The server has seven docking interfaces with different levels of control over docking parameters and in this study, we used the easy interface. As input, the ligand and target structures were provided in PDB format and the potential binding residues were specified as active residues in the input parameters section and the passive residues were set to be defined automatically around the active residues. For the types of molecules being docked, the "Protein/peptide/ligand" option was selected and the following default parameters were used in the docking parameters section. The number of structures for rigid-body docking is set to 1000 and the number of structures retained for semi-flexible refinement was set to 200. During rigid-body energy minimization, 180 degrees rotation was enabled. In the final refinement stage, water is used as the solvent type. Clustering is performed using a Fraction of Common Contact with the cut-off of 0.6 and the minimum cluster size was set to 4. The orientation of the starting structures was set to be randomized and cross-docking was enabled to perform docking between all combinations in the ensembles of starting structures. On the result page, the top 10 clusters of the docking solutions were ranked according to their HADDOCK score, and their detailed statistics were shown.

HDOCK performs a template-based docking if a template is found through sequence similarity search, otherwise, HDOCKlite [38] a hierarchical FFT-based approach, is employed for docking. HDOCKlite employs the iterative knowledge-based scoring function (ITScorePP). The resulting structures are clustered based on backbone RMSD (cut-off of 5 Å). As input, the ligand and the target structures in PDB format were submitted and the potential binding residues were specified but no distance restraints between the potential binding residues are defined. By default, the hybrid protocol of template-based modeling and free docking was performed and no data was provided for the small-angle X-ray scattering experimental data which assists ranking procedure. On the result page, the summary of ranking and docking scores for the top 10 binding modes as well as information about the templates used for target and ligand modeling were provided.

HPEPDOCK is an ensemble docking method where an ensemble of peptide conformations is generated by MODPEP [39] program are docked into the receptor using a rigid docking method, MDOCK [40]. MDOCK employs the iterative knowledge-based scoring function (ITScore). The resulting structures are energy minimized by the SIMPLEX algorithm. The ligand and the target structures in PDB format were provided as input and the binding site was defined by potential binding residue on the target instead of providing reference ligand structure. By default, HPEPDOCK performed ensemble docking of peptide structures generated prior to docking which was set to 1000 to consider peptide flexibility instead of rigid docking with the input peptide conformation. The number of peptide binding modes to output was 100 and the server provided the summary of rankings and docking scores for the top 10 binding modes on the result page.

PatchDock is a shape complementary-based rigid body docking method where a sparse surface of each molecule is divided into geometric patches. The patches of ligand and receptor are matched based on local geometric complementarity using the Geometric Hashing method and the resulting structures are further structurally clustered. Docked poses that have steric clashes with the receptor are excluded and the remaining poses are retained for geometric scoring (see also **Table S17** for more details). To speed up the scoring procedure, ligand poses are scored first with the low-density molecular surface using the sparse surface representation, and the high-scoring 500 poses are re-ranked with the high-density Connolly surface. As input, the ligand and the target structures in PDB format were submitted and the potential binding residues were provided as text files for the target and the ligand but distance constraints between the residues were not specified. The default settings were retained for the clustering tolerance (RMSD of 4 Å) and the complex type (default configuration). As a result, up to 100 peptide binding modes were generated and on the result page, their docking score, desolvation energy, the interface area size, and the actual rigid transformation of the solution were shown.

PEP-FOLD3 considers peptides as a series of four amino acid long fragments, with three overlapping residues. PEP-FOLD3 uses the 27 states of a Hidden Markov Model-derived structural alphabet to determine geometric descriptors of the fragments predicted by a Support Vector Machine. Next, a rigid

assembly of the prototype fragments is performed by Forward Backtrack algorithm followed by Monte-Carlo refinement and structural clustering. As input, the target structure in PDB format and the peptide sequence in FASTA format were provided. The binding site was defined as the potential binding residue on the target. To avoid biased modeling, no reference ligand structure was provided. The default settings were used for the number of simulations (100), the scoring (sOPEP), and the number of Monte Carlo steps (30000). The result page of PEP-FOLD3 provides the conformations and statistics of the top 5 clusters of the docked poses ranked according to their sOPEP score.

PIPER-FlexPepDock docks an ensemble of peptide conformations that are extracted as short fragments from the PDB by the Rosetta fragment picker [41], as rigid bodies onto the receptor using PIPER docking [37] followed by a single run of fully flexible refinement by the Rosetta Flex-PepDock Refinement algorithm [42]. The resulting docked poses are clustered. As input, the target structure in PDB format and the peptide sequence in one-letter format was provided. Receptor flexibility mode is on by default and to avoid bias in the fragment selection step, no secondary structures were defined for peptide. As a result, the top 10 binding modes and their re-weight and interface scores were provided on the results page.

References

1. Bortoluzzi, A.; Amato, A.; Lucas, X.; Blank, M.; Ciulli, A. Structural Basis of Molecular Recognition of Helical Histone H3 Tail by PHD Finger Domains. *Biochemical Journal* **2017**, *474*, 1633–1651, doi:10.1042/BCJ20161053.
2. Alexander J Ruthenburg, Wooikoon Wang, Daina M Graybosch, Haitao Li, C.D.; Allis, Dinshaw J Patel, and G.L.V. Histone H3 Recognition and Presentation by the WDR5 Module of the MLL1 Complex. *Nat Struct Mol Biol* **2006**, *13*, 704–712, doi:10.1016/j.physbeh.2017.03.040.
3. Ooi, S.K.T.; Qiu, C.; Bernstein, E.; Li, K.; Jia, D.; Yang, Z.; Erdjument-Bromage, H.; Tempst, P.; Lin, S.P.; Allis, C.D.; et al. DNMT3L Connects Unmethylated Lysine 4 of Histone H3 to de Novo Methylation of DNA. *Nature* **2007**, *448*, 714–717, doi:10.1038/nature05987.
4. Iwase, S.; Xiang, B.; Ghosh, S.; Ren, T.; Lewis, P.W.; Cochrane, J.C.; Allis, C.D.; Picketts, D.J.; Patel, D.J.; Li, H.; et al. ATRX ADD Domain Links an Atypical Histone Methylation Recognition Mechanism to Human Mental-Retardation Syndrome. *Nat Struct Mol Biol* **2011**, *18*, 769–776, doi:10.1038/nsmb.2062.
5. Rajakumara, E.; Wang, Z.; Ma, H.; Hu, L.; Chen, H.; Lin, Y.; Guo, R.; Wu, F.; Li, H.; Lan, F.; et al. PHD Finger Recognition of Unmodified Histone H3R2 Links UHRF1 to Regulation of Euchromatic Gene Expression. *Mol Cell* **2011**, *43*, 275–284, doi:10.1016/j.molcel.2011.07.006.
6. Tsai, W.W.; Wang, Z.; Yiu, T.T.; Akdemir, K.C.; Xia, W.; Winter, S.; Tsai, C.Y.; Shi, X.; Schwarzer, D.; Plunkett, W.; et al. TRIM24 Links a Non-Canonical Histone Signature to Breast Cancer. *Nature* **2010**, *468*, 927–932, doi:10.1038/nature09542.
7. Chignola, F.; Gaetani, M.; Rebane, A.; Org, T.; Mollica, L.; Zucchelli, C.; Spitaleri, A.; Mannella, V.; Peterson, P.; Musco, G. The Solution Structure of the First PHD Finger of Autoimmune Regulator in Complex with Non-Modified Histone H3 Tail Reveals the Antagonistic Role of H3R2 Methylation. *Nucleic Acids Res* **2009**, *37*, 2951–2961, doi:10.1093/nar/gkp166.
8. Org, T.; Chignola, F.; Hetényi, C.; Gaetani, M.; Rebane, A.; Liiv, I.; Maran, U.; Mollica, L.; Bottomley, M.J.; Musco, G.; et al. The Autoimmune Regulator PHD Finger Binds to Non-Methylated Histone H3K4 to Activate Gene Expression. *EMBO Rep* **2008**, *9*, 370–376, doi:10.1038/embor.2008.11.
9. Zhang, Y.; Yang, H.; Guo, X.; Rong, N.; Song, Y.; Xu, Y.; Lan, W.; Zhang, X.; Liu, M.; Xu, Y.; et al. The PHD1 Finger of KDM5B Recognizes Unmodified H3K4 during the Demethylation of Histone H3K4me2/3 by KDM5B. *Protein Cell* **2014**, *5*, 837–850, doi:10.1007/s13238-014-0078-4.
10. Li, H.; Ilin, S.; Wang, W.; Duncan, E.M.; Wysocka, J.; Allis, C.D.; Patel, D.J. Molecular Basis for Site-Specific Read-out of Histone H3K4me3 by the BPTF PHD Finger of NURF. *Nature* **2006**, *442*, 91–95, doi:10.1038/nature04802.

11. Chauvot de Beauchene, I.; de Vries, S.J.; Zacharias, M. Binding Site Identification and Flexible Docking of Single Stranded RNA to Proteins Using a Fragment-Based Approach. *PLoS Comput Biol* **2016**, *12*, 1–21, doi:10.1371/journal.pcbi.1004697.
12. Liao, J.M.; Wang, Y.T.; Lin, C.L.S. A Fragment-Based Docking Simulation for Investigating Peptide-Protein Bindings. *Physical Chemistry Chemical Physics* **2017**, *19*, 10436–10442, doi:10.1039/c6cp07136h.
13. Budin, N.; Majeux, N.; Caflisch, A. Fragment-Based Flexible Ligand Docking by Evolutionary Optimization. *Biol Chem* **2001**, *382*, 1365–1372, doi:10.1515/BC.2001.168.
14. Zsoldos, Z.; Reid, D.; Simon, A.; Sadjad, S.B.; Johnson, A.P. EHiTS: A New Fast, Exhaustive Flexible Ligand Docking System. *J Mol Graph Model* **2007**, *26*, 198–212, doi:10.1016/j.jmgm.2006.06.002.
15. Thompson, D.C.; Denny, R.A.; Nilakantan, R.; Humblet, C.; Joseph-McCarthy, D.; Feyfant, E. CONFIRM: Connecting Fragments Found in Receptor Molecules. *J Comput Aided Mol Des* **2008**, *22*, 761–772, doi:10.1007/s10822-008-9221-8.
16. Samsonov, S.A.; Zacharias, M.; Chauvot de Beauchene, I. Modeling Large Protein–Glycosaminoglycan Complexes Using a Fragment-Based Approach. *J Comput Chem* **2019**, *40*, 1429–1439, doi:10.1002/jcc.25797.
17. Bálint, M.; Horváth, I.; Mészáros, N.; Hetényi, C. Towards Unraveling the Histone Code by Fragment Blind Docking. *Int J Mol Sci* **2019**, *20*, 422, doi:10.3390/ijms20020422.
18. Cross, S.S.J. Improved FlexX Docking Using FlexS-Determined Base Fragment Placement. *J Chem Inf Model* **2005**, *45*, 993–1001, doi:10.1021/ci050026f.
19. Morris, G.M.; Huey, R.; Lindstrom, W.; Sanner, M.F.; Belew, R.K.; Goodsell, D.S.; Olson, A.J. AutoDock4 and AutoDockTools4: Automated Docking with Selective Receptor Flexibility. *J Comput Chem* **2009**, *30*, 2785–2791, doi:10.1002/jcc.21256.
20. Kurcinski, M.; Jamroz, M.; Blaszczyk, M.; Kolinski, A.; Kmiecik, S. CABS-Dock Web Server for the Flexible Docking of Peptides to Proteins without Prior Knowledge of the Binding Site. *Nucleic Acids Res* **2015**, *43*, W419–W424, doi:10.1093/nar/gkv456.
21. Kozakov, D.; Hall, D.R.; Xia, B.; Porter, K.A.; Padhorny, D.; Yueh, C.; Beglov, D.; Vajda, S. The ClusPro Web Server for Protein–Protein Docking. *Nat Protoc* **2017**, *12*, 255–278, doi:10.1038/nprot.2016.169.
22. Tovchigrechko, A.; Vakser, I.A. GRAMM-X Public Web Server for Protein-Protein Docking. *Nucleic Acids Res* **2006**, *34*, W310–W314, doi:10.1093/nar/gkl206.
23. van Zundert, G.C.P.; Rodrigues, J.P.G.L.M.; Trellet, M.; Schmitz, C.; Kastritis, P.L.; Karaca, E.; Melquiond, A.S.J.; van Dijk, M.; de Vries, S.J.; Bonvin, A.M.J.J. The HADDOCK2.2 Web Server:

User-Friendly Integrative Modeling of Biomolecular Complexes. *J Mol Biol* **2016**, *428*, 720–725, doi:10.1016/j.jmb.2015.09.014.

24. Schneidman-Duhovny, D.; Inbar, Y.; Nussinov, R.; Wolfson, H.J. PatchDock and SymmDock: Servers for Rigid and Symmetric Docking. *Nucleic Acids Res* **2005**, *33*, W363–W367, doi:10.1093/nar/gki481.
25. Lamiable, A.; Thévenet, P.; Rey, J.; Vavrusa, M.; Derreumaux, P.; Tufféry, P. PEP-FOLD3: Faster de Novo Structure Prediction for Linear Peptides in Solution and in Complex. *Nucleic Acids Res* **2016**, *44*, W449–W454, doi:10.1093/nar/gkw329.
26. Zhou, P.; Jin, B.; Li, H.; Huang, S.-Y. HPEPDOCK: A Web Server for Blind Peptide–Protein Docking Based on a Hierarchical Algorithm. *Nucleic Acids Res* **2018**, *46*, W443–W450, doi:10.1093/nar/gky357.
27. Yan, Y.; Zhang, D.; Zhou, P.; Li, B.; Huang, S.-Y. HDOCK: A Web Server for Protein–Protein and Protein–DNA/RNA Docking Based on a Hybrid Strategy. *Nucleic Acids Res* **2017**, *45*, W365–W373, doi:10.1093/nar/gkx407.
28. Kozakov, D.; Brenke, R.; Comeau, S.R.; Vajda, S. PIPER: An FFT-Based Protein Docking Program with Pairwise Potentials. *Proteins: Structure, Function, and Bioinformatics* **2006**, *65*, 392–406, doi:10.1002/prot.21117.
29. Waterhouse, A.; Bertoni, M.; Bienert, S.; Studer, G.; Tauriello, G.; Gumienny, R.; Heer, F.T.; de Beer, T.A.P.; Rempfer, C.; Bordoli, L.; et al. SWISS-MODEL: Homology Modelling of Protein Structures and Complexes. *Nucleic Acids Res* **2018**, *46*, W296–W303, doi:10.1093/nar/gky427.
30. Consortium, T.U. UniProt: A Worldwide Hub of Protein Knowledge. *Nucleic Acids Res* **2019**, *47*, D506–D515, doi:10.1093/nar/gky1049.
31. Rackers, J.A.; Wang, Z.; Lu, C.; Laury, M.L.; Lagardère, L.; Schnieders, M.J.; Piquemal, J.-P.; Ren, P.; Ponder, J.W. Tinker 8: Software Tools for Molecular Design. *J Chem Theory Comput* **2018**, *14*, 5273–5289, doi:10.1021/acs.jctc.8b00529.
32. Wang, J.; Wolf, R.M.; Caldwell, J.W.; Kollman, P.A.; Case, D.A. Development and Testing of a General Amber Force Field. *J Comput Chem* **2004**, *25*, 1157–1174, doi:10.1002/jcc.20035.
33. Gasteiger, J.; Marsili, M. Iterative Partial Equalization of Orbital Electronegativity—a Rapid Access to Atomic Charges. *Tetrahedron* **1980**, *36*, 3219–3228, doi:10.1016/0040-4020(80)80168-2.
34. Hetényi, C.; Van Der Spoel, D. Blind Docking of Drug-Sized Compounds to Proteins with up to a Thousand Residues. *FEBS Lett* **2006**, *580*, 1447–1450, doi:10.1016/j.febslet.2006.01.074.
35. Kolinski, A. Protein Modeling and Structure Prediction with a Reduced Representation. *Acta Biochim Pol* **2004**, *51*, 349–371.

36. Shen, M.; Sali, A. Statistical Potential for Assessment and Prediction of Protein Structures. *Protein Science* **2006**, *15*, 2507–2524, doi:10.1110/ps.062416606.
37. Kozakov, D.; Brenke, R.; Comeau, S.R.; Vajda, S. PIPER: An FFT-Based Protein Docking Program with Pairwise Potentials. *Proteins: Structure, Function, and Bioinformatics* **2006**, *65*, 392–406, doi:10.1002/prot.21117.
38. Huang, S.-Y.; Zou, X. MDockPP: A Hierarchical Approach for Protein-Protein Docking and Its Application to CAPRI Rounds 15-19. *Proteins: Structure, Function, and Bioinformatics* **2010**, *78*, 3096–3103, doi:10.1002/prot.22797.
39. Yan, Y.; Zhang, D.; Huang, S.-Y. Efficient Conformational Ensemble Generation of Protein-Bound Peptides. *J Cheminform* **2017**, *9*, 59, doi:10.1186/s13321-017-0246-7.
40. Huang, S.-Y.; Zou, X. Ensemble Docking of Multiple Protein Structures: Considering Protein Structural Variations in Molecular Docking. *Proteins: Structure, Function, and Bioinformatics* **2006**, *66*, 399–421, doi:10.1002/prot.21214.
41. Gront, D.; Kulp, D.W.; Vernon, R.M.; Strauss, C.E.M.; Baker, D. Generalized Fragment Picking in Rosetta: Design, Protocols and Applications. *PLoS One* **2011**, *6*, e23294, doi:10.1371/journal.pone.0023294.
42. Raveh, B.; London, N.; Schueler-Furman, O. Sub-Angstrom Modeling of Complexes between Flexible Peptides and Globular Proteins. *Proteins: Structure, Function, and Bioinformatics* **2010**, *78*, 2029–2040, doi:10.1002/prot.22716.
